# Innovative Castor Oil Derivative Synthesized through a Sustainable Approach Generating Reactive Cross-Linker from Secondary Products for Additive Manufacturing

**DOI:** 10.1021/acspolymersau.5c00055

**Published:** 2025-07-30

**Authors:** Vojtěch Jašek, Veronika Lavrinčíková, Otakar Bartoš, Jan Prokeš, Radek Přikryl, Silvestr Figalla

**Affiliations:** Institute of Materials Chemistry, Faculty of Chemistry, 48274 Brno University of Technology, 61200 Brno, Czech Republic

**Keywords:** castor oil, methacrylate, biobased, curable resin, transesterification, additive manufacturing

## Abstract

Additive manufacturing utilizes various reactive precursors to fabricate diverse products, including prototypes, functional components, and designer objects. This work presents a synthesis approach toward a novel biobased printable compound, 2-hydroxypropyl ricinoleate dimethacrylate (2-HPRDM). Our proposed strategy involves the castor oil transesterification process, producing 2-hydroxypropyl ricinoleate (2-HPR). We used high-performance liquid chromatography (HPLC) analysis to investigate the reaction progress at equimolar and excess reactant concentrations. This fatty acid ester was modified with methacrylic anhydride to form 2-HPRDM, releasing the secondary reaction product methacrylic acid (MA). This compound was used for the synthesis of propylene glycol dimethacrylate (PGDMA), which valorized all potential wastes generated during the 2-HPRDM production. This article presents the innovative vacuum-distillation esterification approach that generates PGDMA. All synthesized compounds were structurally characterized via NMR, ESI-MS, and FTIR analyses. The formed curable compounds were fabricated into testing specimens and a detailed prototype by an mSLA three-dimensional (3D) printer to confirm their usability. The 3D-printed object was used for the mechanical and thermomechanical characterization of the formulated curable resins via dynamic mechanical analysis (DMA), tensile, and flexural tests. The best-performing 2-HPRDM-based system contained 45 wt % of PGDMA and recorded a storage modulus of 750 MPa, a glass-transition temperature of 85.6 °C, a cross-linking density of 18.9 kmol/m^3^, a tensile strength of 16.1 ± 2.4 MPa, and a flexural strength of 14.3 ± 1.0 MPa.

## Graphical abstract

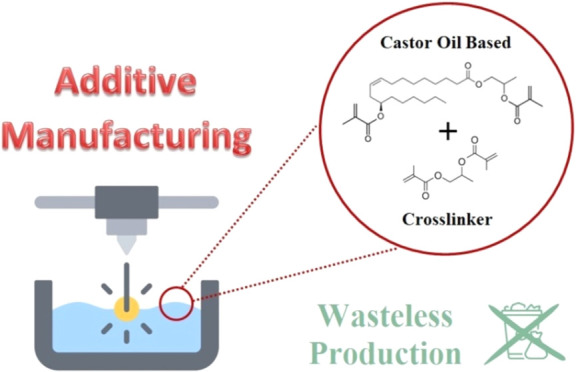

## 1 Introduction

Castor oil is one of the most widely used triacylglycerides among the known vegetable-based lipids for numerous material applications.^1–4^ This natural product exhibits a specific molecular structure differing from those of other vegetable oils. Castor oil contains primarily ricinoleic acid out of all existing oil-forming fatty acids (the content varies typically from 85 to 90%).^5,6^ This unique triacylglyceride has potential applications in various fields, including cosmetics,^7^ pharmaceuticals,^8^ lubricants,^9^ adhesives,^10^ coatings,^11^ biomaterials,^12^ and inks and paints.^13^ The presence of a vacant hydroxyl group bonded on C12 carbon combined with the unsaturated bond between C9 and C10 carbons ensures the exceptional reactivity toward potential nucleophilic substitutions leading to the formation of esters, ethers, or urethanes^14^ while maintaining the liquid state at moderate temperatures due to the van der Waals force suppression caused by the determined molecules’ steric state by the unsaturated double bond.^15^ Moreover, the unsaturated bonding may be modified using several chemical approaches, such as epoxidation,^16^ thiol–ene addition,^17^ or Diels–Alder reactions.^18^ These structural characteristics provide promising utility potential in sustainable, high-renewable-carbon-content material fields focused on the substitution of fossil-based compounds.

In the available literature sources, additive manufacturing is frequently linked to vegetable oil-based reactive curable resins, representing a sustainable alternative to precursors from nonrenewable sources.^35–38^ Several chemical approaches are employed for generating curable resins suitable for photoinitiation polymerization of thermosets. Vegetable oil epoxidation is one of the most widely used, investigated, and well-described production pathways.^19–22^ Many different epoxidizing systems were used, namely, on *meta*-chloroperbenzoic acid (mCPBA)^19^ or hydrogen peroxide (H_2_O_2_) in combination with formic acid,^20^ acetic acid,^21^ or mineral acids.^22^ Thiol–ene additions were also numerously investigated in the published articles.^23,24^ This modification concept describes unsaturated double bond modification via addition using sulfur-containing compounds. Mercaptoethanol and thioglycolic acid are the most commonly used molecules containing the −SH functional group capable of reacting with double bonds within the vegetable oil’s carbon backbone.^23,24^ Epoxidation ensures the presence of the highly reactive epoxy functional groups undergoing nucleophilic substitutions with numerous compounds leading either to the intermediates (reaction with anhydrides, water, or alcohols)^25–27^ or directly to produce acrylate, methacrylate, or itaconate derivatives suitable for three-dimensional (3D) printing.^28–30^ The intermediate production from epoxidized vegetable oils and the triacylglycerides modified via thiol–ene additions typically contain multiple modifiable functional groups (hydroxyls or carboxyls), serving further nucleophilic substitution, generating polymerizable precursors.^23,26^ Multiple nucleophiles ensuring the triacylglyceride’s curability are used, namely, acyl halides,^31^ anhydrides,^32^ or particular carboxylic acids.^30^

Castor oil contains both unsaturated double bonds and naturally occurring free hydroxyl functional groups. Therefore, this triacylglyceride may be premodified using previously described concepts or direct esterification may occur to avoid additional synthesis and purification steps. Several published works focus on the polyurethane-forming castor oil-based materials.^33,34^ Particular publications describe the direct acrylate modification of castor oil, appropriate for radical polymerization initiated by visible light, which is the optimal compound composition for SLA and DLP 3D printing. The reported investigations described the free hydroxyls modified by acryloyl chloride;^35^ particular isocyanate-acrylate/methacrylate compounds were used for the urethane-bonded acrylates (isophorone diisocyanate (IPDI) or 2-(*tert*-butylamino)­ethyl methacrylate (TBEM)),^36,37^ or methacrylic anhydride.^38,41^ Typically, vegetable oil-based curable precursors possess high viscosity and insufficient mechanical strength for rigid thermosets due to the long carbon chains in ricinoleic acid.^39,40^ The reported tensile strengths of modified castor oil-based precursors reached low values (3.2 ± 0.2 MPa or up to 0.8 MPa).^39,40^

This work aims to propose a new castor oil-based derivative, 2-hydroxypropyl ricinoleate (2-HPR), produced via castor oil transesterification in the presence of excess propylene glycol using sodium hydroxide as an available and efficient catalyst. After the reaction, the propylene glycol excess was distilled and the formed 2-HPR was purified. This novel ricinoleic acid derivative contains two modifiable hydroxyl groups (see Figure 1). We esterified the hydroxyl groups (reactive nucleophiles) using methacrylic anhydride (MAAH) as a reactive acyl derivative and potassium acetate as a sustainable and inexpensive catalyst. During the methacrylation with MAAH, the secondary product, methacrylic acid (MA), is formed. We quantitatively distilled the formed MA to increase process sustainability and obtain an added-value compound. Eventually, we valorized the distilled propylene glycol excess from the transesterification and the methacrylic acid formed during 2-HPR methacrylation via direct Fischer esterification, producing propylene glycol dimethacrylate (PGDMA). The modified 2-HPR and the produced PGDMA from the secondary products were used to form a curable reactive mixture generated via a wasteless approach used for additive manufacturing. Our investigation reports the synthesis of a novel biobased reactive compound produced by a sustainable, wasteless concept.

**Figure 1:**
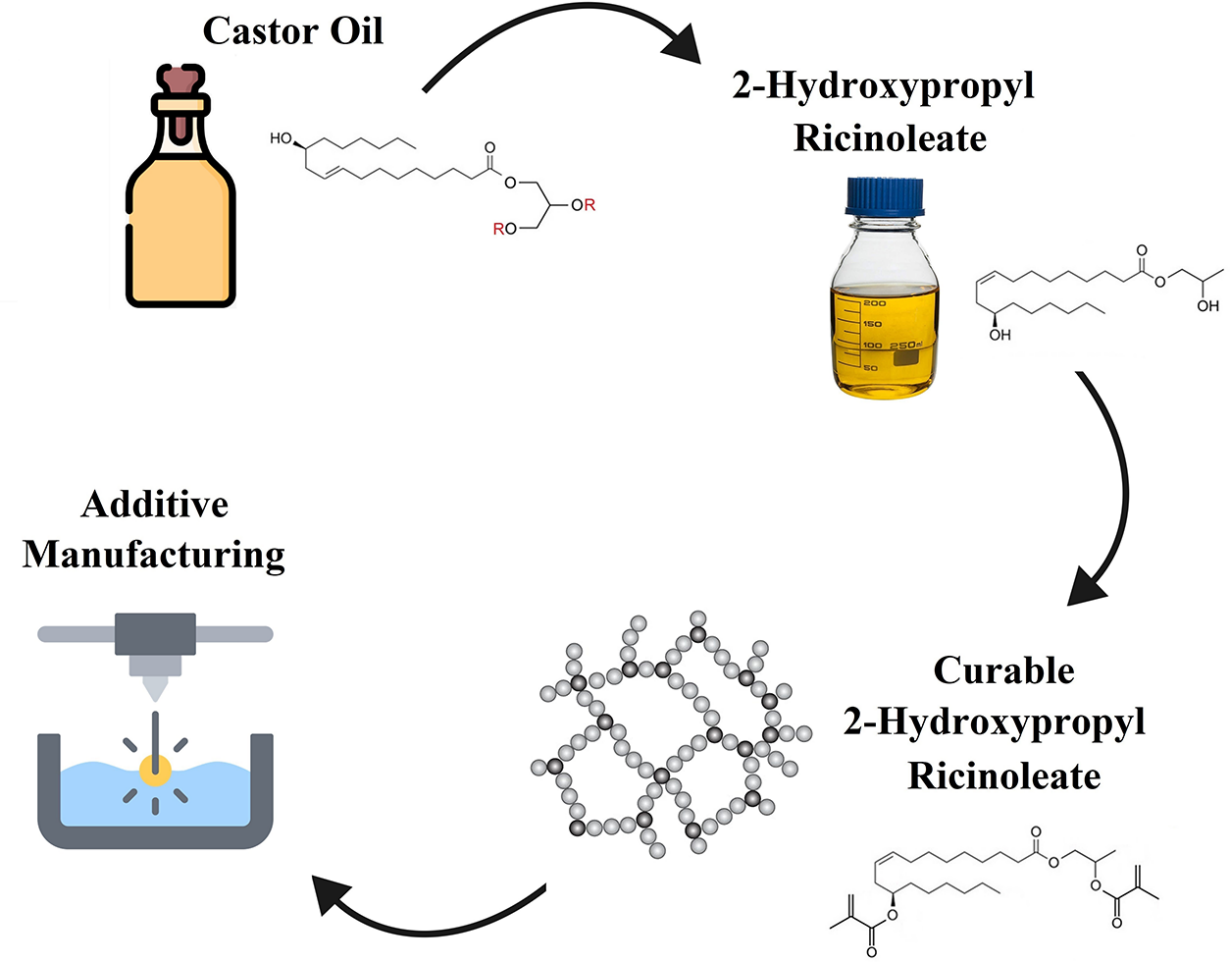
Proposed strategy for the production of 2-hydroxypropyl ricinoleate dimethacrylate for additive manufacturing (recreated illustrations with permission following *Flaticon License*).

## 2 Experimental Section

### 2.1 Materials

Castor oil (90 wt % of ricinoleic acid, declared by the supplier) and propylene glycol (99%) for 2-hydroxypropyl ricinoleate synthesis were kindly acquired from Fichema Ltd. (Czech Republic). Sodium hydroxide (p.a., transesterification catalyst), potassium acetate (99%, methacrylation catalyst), sodium sulfate (99%, anhydrous, drying agent), ethyl acetate (99%, extraction solvent), sulfuric acid (98%, esterification catalyst), and chloroform (99%, stabilized by ethanol, HPLC mobile phase) were obtained from PENTA Chemicals Ltd. (Czech Republic). Methacrylic anhydride (98%, nucleophile for the methacrylation), 4-methoxyphenol (99%, spontaneous polymerization inhibitor), BAPO photoinitiator (phenylbis­(2,4,6-trimethylbenzoyl)­phosphine oxide, 97%) for the specimen preparation, and chloroform-d (CDCl_3_, 99.8% D atom) for the NMR analysis were all purchased from Merck Life Science Ltd. (Czech Republic).

### 2.2 Products’ Analysis

High-performance liquid chromatography (HPLC) was used for the castor oil transesterification investigation. We used HPLC instrumentation (Agilent 1100, CA) in chloroform (CHCl_3_). The measured samples were prepared by diluting 3 mg of the analyzed reaction mixture in 1 mL of the mobile phase. The measured solutions were filtered through a 0.22 μm PTFE filter to remove solid particles. The analysis parameters were as follows: mobile phase flow of 1 mL/min (isocratic elution); column temperature of 30 °C; used column: Ascentis C18 (5 μm) (L × I.D. 5 cm × 4.6 mm, HPLC Column).

Nuclear magnetic resonance (NMR) was used to obtain the ^1^H spectrum, verifying the synthesized molecules’ chemical structures. A Bruker Avance III 500 MHz (Bruker, Billerica, MA) with a measuring frequency of 500 MHz for ^1^H NMR at 30 °C provided the analyses. d-chloroform (CDCl_3_) served as a solvent, and tetramethylsilane (TMS) served as an internal standard. The chemical shifts (δ) are expressed in parts per million (ppm) units referenced by a solvent. Coupling constant *J* has (Hz) unit with coupling expressed as s-singlet, d-doublet, t-triplet, q-quartet, p-quintet, and m-multiplet.

Fourier-transform infrared spectroscopy (FTIR) served as another structural confirmation method. A Bruker Tensor 27 (Billerica, MA) was used for all of the measurements, and the attenuated total reflectance (ATR) method was applied, where a diamond was a dispersion component. The diode laser served as an irradiation source. A Michelson interferometer eventually quantified the measured signal. All illustrated spectra were composed of 32 total scans with a resolution of 2 cm^–1^.

The molecular structure was also verified by mass spectrometry (MS) (Bruker EVOQ LC-TQ) using electrospray ionization (ESI). Product scan spectra were obtained by fragmentation of the following: precursor ions [M + H *–* H_2_O]+: *m*/*z* 338.9 (2-HPR) and *m*/*z* 475.2 (2-HPR MMA); precursor ion [M *–* H]^−^: *m*/*z* 85.1 (MA); precursor ion [M + H]^+^: *m*/*z* 211.8 (PGDMA). Collision energy spread (5 – 20 eV) improved the collected MS/MS data quality. Furthermore, the obtained mass spectra agree with their in silico prediction by CFM-ID 4.0,^42^ which also proposed the product ion structure for the most intense masses.

### 2.3 Castor Oil Transesterification with Propylene Glycol

Castor oil (500 g, 0.54 equiv) was mixed with propylene glycol in two ratios with respect to the ester bonds in the triacylglyceride: the equimolar (123 g, 1.62 equiv) and the excess (246 g, 3.24 equiv). The reaction mixture was heated to a reflux temperature (188 °C), and then, the catalyst (NaOH, 0.5 g, 0.0125 equiv) was introduced into the refluxing solution. The HPLC monitored the transesterification progress, which commenced with the addition of the catalyst. The reaction lasted 30 min (see Figure 2), and progress samples were obtained a total of six times during the transesterification. After the reaction, the equimolar post-transesterification mixture was diluted with ethyl acetate (50 vol % of the solvent) and extracted with distilled water once to remove the generated glycerol, residual propylene glycol, and the catalyst. After purification, the diluted reaction mixture was dried over anhydrous sodium sulfate and ethyl acetate was distilled to obtain the pure product. Except for the first purification step, the excess post-transesterification mixture was purified in the same way. The reaction mixture with an excess of propylene glycol was initially neutralized with sulfuric acid to inactivate the alkali catalyst and then distilled to remove all excess propylene glycol from the solution. The distillation was performed at 20 Torr and 90 °C. After the distillation, 2-HPR obtained from the excess transesterification was characterized via ^1^H NMR, ESI-MS, and FTIR.

**Figure 2:**
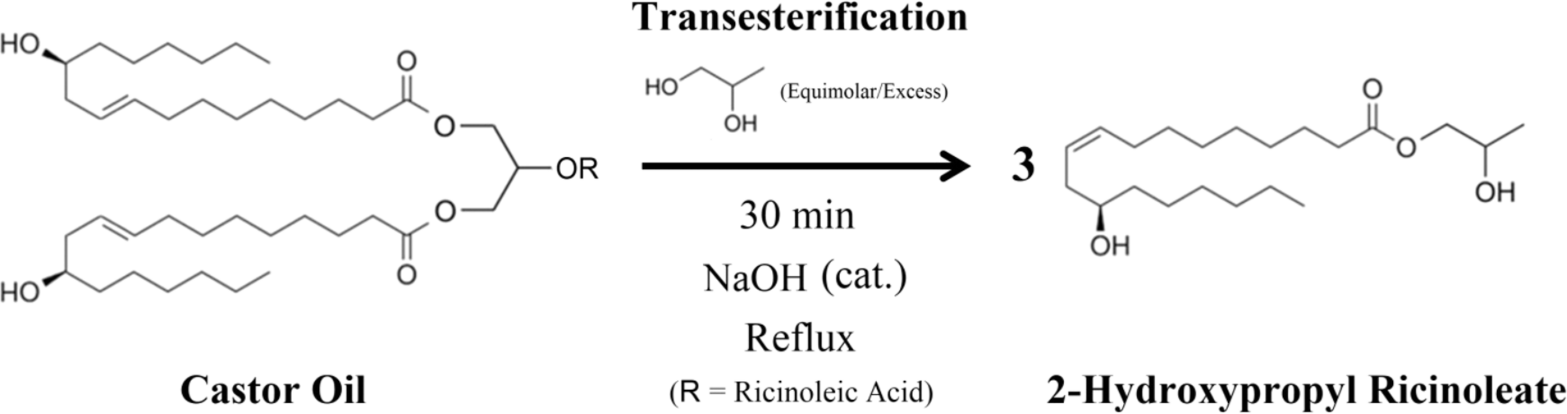
Castor oil transesterification reaction scheme producing 2-hydroxypropyl ricinoleate (2-HPR).

#### 2-Hydroxypropyl Ricinoleate (2-HPR) (Yield: 85.6%)

^1^H NMR (Figure S1) (500 MHz, CDCl_3_): δ 5.60–5.51 (m, 1H), 5.40 (dtt, *J* = 10.9, 7.5, 1.7 Hz, 1H), 4.11 (dd, *J* = 11.1, 3.2 Hz, 1H), 3.93 (dd, *J* = 11.1, 7.3 Hz, 1H), 3.71–3.56 (m, 2H), 2.34 (q, *J* = 7.5 Hz, 2H), 2.24–2.18 (m, 2H), 2.05 (dt, *J* = 7.6, 5.9 Hz, 2H), 1.63 (q, *J* = 2.6 Hz, 2H), 1.47 (t, *J* = 4.1 Hz, 2H), 1.38–1.25 (m, 16H), 1.22 (dd, *J* = 12.1, 6.4 Hz, 3H), 0.94–0.82 (m, 3H).

ESI-MS fragmentation spectrum (Figure S2) and (C_21_H_40_O_4_) spectrum calc. [M+H*–*H_2_O]^+^ 339.5 *m*/*z*, found 338.9 *m*/*z*.

FTIR spectra (Figure S3) with absorption wavenumber intervals: O–H stretch. 3550–3200 cm^–1^, C:H stretch, 3000–2840 cm^–1^, C=O (ester) stretch, 1750–1735 cm^–1^, C=C stretch, 1662–1626 cm^–1^, C:O (ester) stretch, 1210–1163 cm^–1^, C=C bend, 840–790 cm^–1^.

### 2.4 2-Hydroxypropyl Ricinoleate Methacrylation

The previously synthesized 2-hydroxypropyl ricinoleate (340 g, 1 equiv) was transferred into a 1000 mL three-necked round-bottom flask. Methacrylic anhydride (308 g, 2 equiv) was then added and mixed with 2-HPR. Potassium acetate (6 g, 0.06 equiv) was added to the reaction mixture. The methacrylation lasted 24 h and occurred at 70 °C. After the reaction time, the formed byproduct, methacrylic acid (MA), was distilled from the reaction system to increase the process’s sustainability and to obtain a polymerizable carboxylic acid for further Fisher esterification. The distillation was performed at a reduced pressure (75 Torr) at 100 °C with continuous airflow (20 mL of air per minute), which increased the volatility of the distilled compound and inhibited the spontaneous polymerization. After the separation of MA, the synthesized 2-hydroxypropyl ricinoleate dimethacrylate (2-HPRDM) was washed with distilled water to remove the catalyst and then dried over anhydrous sodium sulfate. The distilled MA and the purified 2-HPRDM were analyzed via ^1^H NMR, ESI-MS, and FTIR to confirm their molecular structure.

#### 2-Hydroxypropyl Ricinoleate Dimethacrylate (2-HPRDMA) (Yield: 94.5%)

^1^H NMR (Figure S4) (500 MHz, CDCl_3_) δ 6.31–6.00 (m, 2H), 5.84–5.46 (m, 2H), 5.49–4.75 (m, 3H), 4.43–4.01 (m, 3H), 2.39–2.17 (m, 4H), 2.11–1.88 (m, 6H), 1.64–1.47 (m, 4H), 1.46–1.19 (m, 21H), 0.92–0.84 (m, 3H).

ESI-MS fragmentation spectrum (Figure S5) and (C_29_H_48_O_6_) spectrum calc. [M+H*–*H_2_O]^+^ 475.7 *m*/*z*, found 475.2 *m*/*z*.

FTIR spectra (Figure S6) with absorption wavenumber intervals: C–H stretch. 3000–2840 cm^–1^, C=O (ester) stretch. 1750–1735 cm^–1^, C=C stretch. 1662–1626 cm^–1^, C–O (ester) stretch. 1210–1163 cm^–1^, C=C bend. 840–790 cm^–1^.

#### Methacrylic Acid (MA) (Yield: 92.4%)

^1^H NMR (Figure S7) (500 MHz, CDCl_3_) δ: 11.68 (s, 1H), 6.26 (dd, *J* = 1.5, 1.0 Hz, 1H), 5.68 (p, *J* = 1.6 Hz, 1H), 1.96 (dd, *J* = 1.6, 1.0 Hz, 3H).

ESI-MS (Figure S8) fragmentation spectrum (C_4_H_6_O_2_) spectrum calc. [M–H]^−^ 85.1 *m*/*z*, found 85.1 *m*/*z*.

FTIR spectrum (Figure S9) with absorption wavenumber intervals: O–H stretch. 3550–3200 cm^–1^, C–H stretch, 3000–2840 cm^–1^, C=C stretch, 1662–1626 cm^–1^, C–O (acid) stretch, 1210–1163 cm^–1^, C=C bend, 840–790 cm^–1^.

### 2.5 Propylene Glycol Dimethacrylate Synthesis from Byproducts

The distilled propylene glycol excess (100 g, 1.3 equiv) was added to a 500 mL round-bottom flask, and the distilled byproduct from the methacrylation, methacrylic acid (247 g, 2.86 equiv), was poured into the reaction mixture. The spontaneous polymerization inhibitor, 4-methoxyphenol (1.24 g, 0.01 equiv), was homogenized with the solution, and the mixture was heated to 110 °C. Sulfuric acid (0.98 g, 0.01 equiv) was introduced into the solution. The complete mixture was dehydrated in a custom column-involved apparatus displayed in Figure 5. The reaction water was quantitatively distilled at a pressure of 120 Torr and a temperature of 110 °C. The distilled water and the decreasing acid number were monitored every 30 min during the reaction. Once the reaction water was collected and quantified, the postreaction mixture was washed with distilled water once to remove the catalyst and the polymerization inhibitor. The produced propylene glycol dimethacrylate (PGDMA) was analyzed via ^1^H NMR, ESI-MS, and FTIR to confirm the proposed chemical structure.

#### Propylene Glycol Dimethacrylate (PGDMA) (Yield: 98.5%)

^1^H NMR (Figure S10) (500 MHz, CDCl_3_) δ 6.19–6.01 (m, 2H), 5.56 (dt, *J* = 5.4, 1.6 Hz, 2H), 5.30–5.19 (m, 1H), 4.33–4.02 (m, 2H), 2.01–1.85 (m, 6H), 1.32 (d, *J* = 6.6 Hz, 3H).

ESI-MS (Figure S11) fragmentation spectrum (C_11_H_16_O_4_) spectrum calc. [M + H]^+^ 212.2 *m*/*z*, found 211.8 *m*/*z*.

FTIR spectrum (Figure S12) with absorption wavenumber intervals: C–H stretch. 3000–2840 cm^–1^, C=O (ester) stretch. 1750–1735 cm^–1^, C=C stretch. 1662–1626 cm^–1^, C–O (ester) stretch. 1210–1163 cm^–1^, C=C bend. 840–790 cm^–1^.

### 2.6 3D Printing of the Products

The testing specimens for mechanical and thermomechanical property investigation were fabricated by a PRUSA SL1S (Prusa Research Ltd., Czech Republic) 3D printer. We prepared mixtures of 2-HPRDMA with different PGDMA contents (0, 15, 30, and 45 wt %). All prepared systems were mixed with 1 wt % of photoinitiator, BAPO. The print settings were as follows: the first layer’s exposure time was 40 s, while the exposure time for all subsequent layers was 20 s. The thickness of the cured layer was 50 μm. The test specimens were printed with automatically generated supports along with a raft to ensure good adhesion of the samples to the build platform. After printing, the samples were cleaned with isopropanol and postcured under a 405 nm LED light for 24 h. We used an EIBOS Oceanus commercial postcuring instrumentation with a 405 nm lamp and an irradiation power of 19 W.

### 2.7 Mechanical and Thermomechanical Properties

The mechanical properties were investigated by tensile and flexural testing. Both tests were performed on a Zwick Z 010 testing machine (ZwickRoell GmbH & Co., Ulm, Germany) equipped with a 500 N load cell. The tensile test was performed according to the CSN EN ISO 527 standard using standardized double-paddle specimens (dogbones 5A) with a 4 × 2 mm^2^ cross-sectional area. The test speed was set to 5 mm·min^–1^. For the three-point flexural test, rectangular specimens with dimensions of 80 × 10 × 4 mm were printed according to the CSN EN ISO 178 standard (by which the test was conducted). The loading nose and support radius were 5 mm with a support span of 64 mm. The test speed was set to 10 mm·min^–1^.

The thermomechanical properties were investigated by DMA 2980 from TA Instruments (New Castle, DE). The tested objects had the following parameters: 50 × 10 × 4 mm^3^. Objects were applied into a dual cantilever attachment, and the parameters of applied deformation were 25 μm amplitude and 1 Hz frequency. The temperature increased from 30 to 120 °C at a rate of 3 °C/min. The storage modulus, loss modulus, and tan δ values were obtained directly from the DMA analysis. The cross-linking density ν_e_ was also calculated for all tested systems to provide information regarding the thermoset molecular structure. The equation for ν_e_ calculation stands as follows^43^

1 $$\upsilon_{e}=\frac{E^{\prime }}{3RT^{\prime }}$$

where ν_e_ refers to the cross-linking density (mol/m^3^); *E*′ is the storage modulus in the rubbery plateau region (*E*′ at *T*_g_ + 40 °C) (Pa); *R* represents the gas constant (*J*/(mol·K)); and *T*′ stands for the thermodynamic temperature in the rubbery plateau region (at *T*_g_ + 40 °C) (K).

## 3 Results and Discussion

### 3.1 Production of Curable Reactive Compounds

The synthesized 2-hydroxypropyl ricinoleate underwent transesterification in two different molar ratios with propylene glycol. Since several published articles working with nucleophilic substitutions observed nonquantitative reactant transformations,^44–46^ we chose the equimolar and 100% excess reaction mixtures containing the exact and 100% excess propylene glycol molar amounts required for triacylglyceride transesterification. The excess propylene glycol ensured quantitative substitution in a shorter reaction time. Additionally, our strategy was either to distill the leftover propylene glycol from the reaction mixture for use in the continual transesterification process or to synthesize a reactive diluent for further additive manufacturing purposes. The HPLC reaction investigation is shown in Figure 6 for the equimolar (Figure 6a) and the 100% excess (Figure 6b) transesterification mixtures.

The transesterification investigations by HPLC show the essential influence of the excess propylene glycol content on the reaction progress. The equimolar reaction mixture (Figure 6a) uncovers the equilibrium state after 30 min of the reaction (no reaction progress occurred between 20 and 30 min). On the other hand, the 100% excess mixture (Figure 6b) reached a better conversion already after 2 min compared to the equimolar reaction. The recorded yield (by weight) of the synthesized 2-hydroxypropyl ricinoleate reached 85.6%, mainly due to the minor other fatty acid contents (approximately 10 wt % according to the supplier) and the losses after the purification extraction of the used neutralized catalyst. The recovered propylene glycol excess from the postreaction mixture yielded 90.5%. The published PLA aminolysis reached quantitative product conversion using 400% reaction excess,^44^ the transesterification of the spent bleaching oil (SBEO) using ethylene glycol as a nucleophile recorded 99.74% conversion at 100% excess (similar results to our study using different entering reactants),^45^ and the dimethyl terephthalate (DMT) transesterification with ethylene glycol ended at around 65% conversion after 4 h with 150% excess of the nucleophile.^46^ The required nucleophile excess for the quantitative reaction conversion is connected with the reaction thermodynamics.^47,48^ Since no secondary product is separated during the introduced published reactions, the synthesizing product is in equilibrium with the reactant, and the reaction cannot proceed further unless a more nucleophile is added.

The Fisher esterification of propylene glycol distilled from the castor oil’s transesterification and methacrylic acid obtained as a secondary product of the 2-hydroxypropyl ricinoleate methacrylation is schematically displayed in Figure 7. We utilized the leftover compounds from 2-HPR production (see Figure 2) and 2-HPRDM synthesis (Figure 3) to generate a known compound, propylene glycol dimethacrylate (PGDMA), employing a sustainable and waste-free approach. Generally, PGDMA is synthesized from propylene oxide and methacrylic anhydride,^49^ or from propylene glycol/2-hydroxypropyl methacrylate and methacrylic anhydride.^50^ Both of these synthetic pathways include hazardous and waste-generating compounds. Our proposed approach (Figure 4) incorporates entirely secondary products, forming only reaction water as a byproduct. Therefore, this concept limits the generation of disposal during the entire process while producing curable compounds suitable for additive manufacturing. The results shown in Figure 7 verify that both indicating parameters, the amount of condensed reaction water and the acid number exhibited by methacrylic acid, progress toward the PGDMA reaction direction. After the reaction time, the total reaction water amount was concluded in a 94.3% yield, and the determined acid number decreased by 94.8% after esterification.

**Figure 3:**
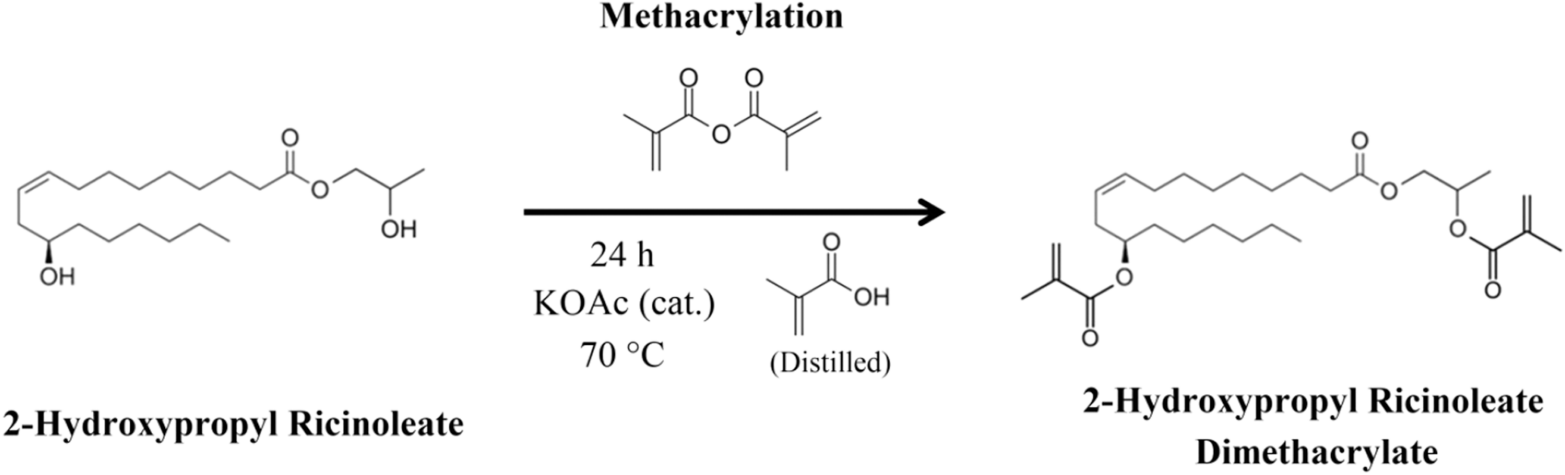
2-Hydroxypropyl ricinoleate methacrylation reaction scheme producing 2-hydroxypropyl ricinoleate dimethacrylate (2-HPRDM).

**Figure 4:**
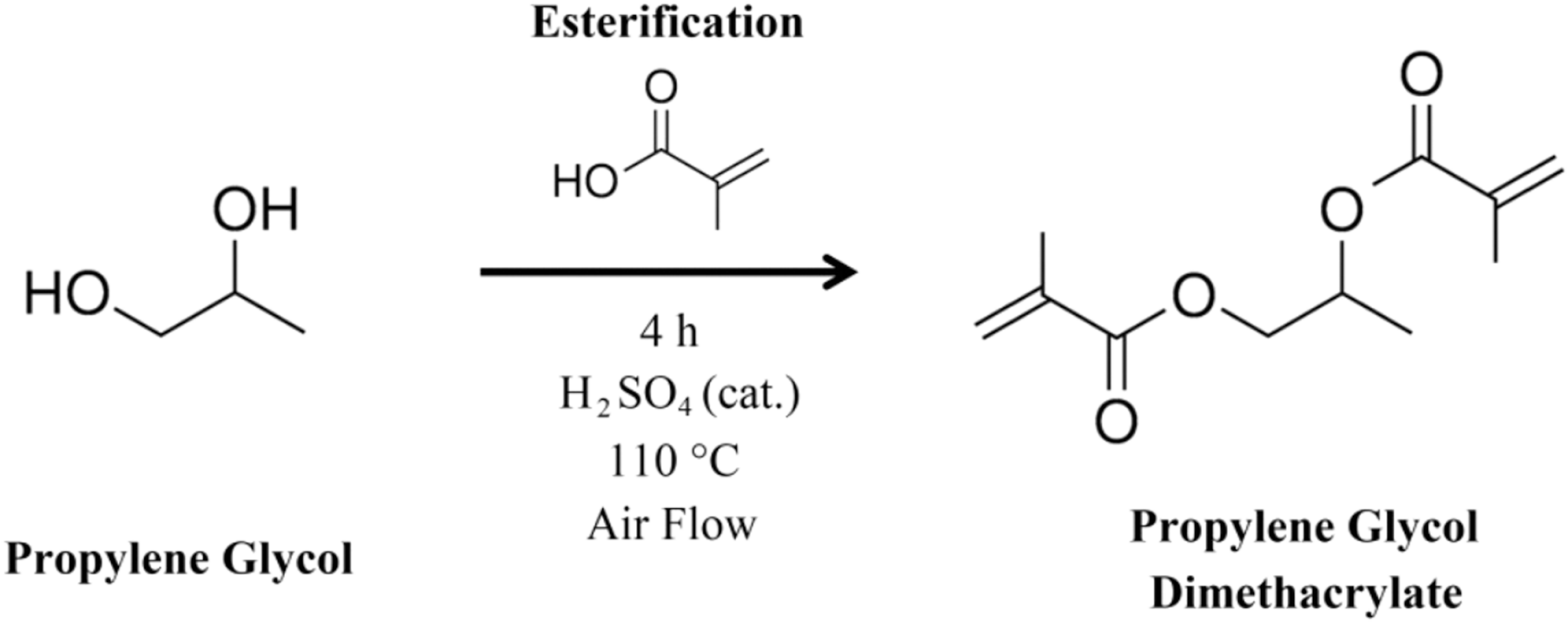
Propylene glycol esterification reaction scheme producing propylene glycol dimethacrylate (PGDMA).

The entire proposed curable castor oil-based derivatives include the water extraction of the transesterification catalyst (neutralized sodium hydroxide), the formed glycerol, potassium acetate (methacrylation catalyst; see Figure 3), and sulfuric acid used for the esterification. Except for the used catalysts and the biobased glycerol, no secondary products were generated during the whole production. Additionally, a custom, novel apparatus for more sustainable Fisher esterification was designed and is described in Figure 5b. This hardware makes esterification much more scalable and efficient as the secondary product is separated, which increases the reaction rate according to the Le Chatelier principle. Additionally, no supportive azeotropic solvent (typically toluene) is needed with a Dean–Stark apparatus.^54,55^ This approach ensures maximum efficiency while generating no VOCs Figures 6 and 7.

**Figure 5:**
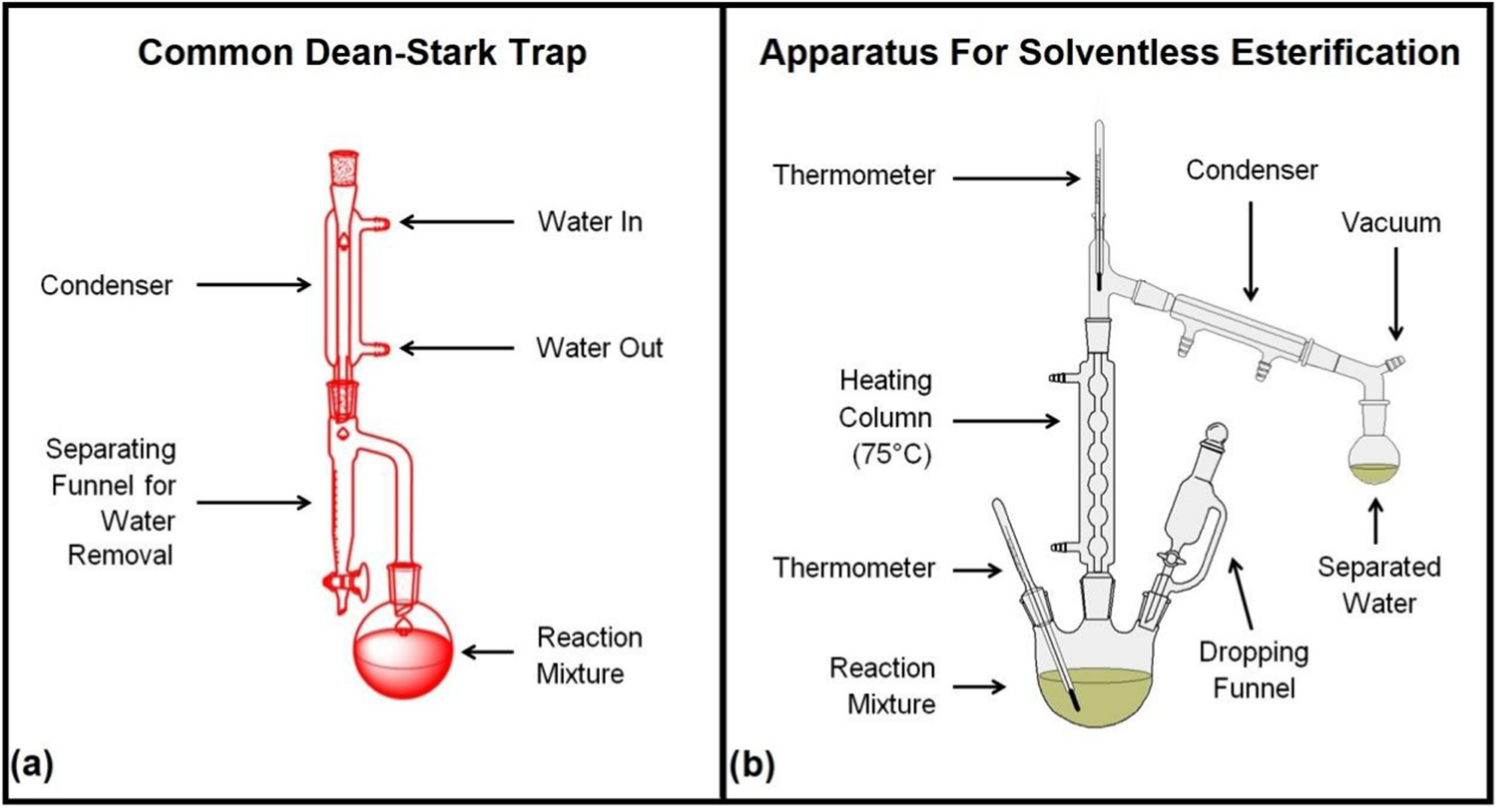
(a) The standard apparatus for the Fisher esterification using azeotropic distillation water removal. (b) The custom apparatus for the Fisher esterification for the solventless water removal used for the propylene glycol dimethacrylate (PGDMA) production.

**Figure 6:**
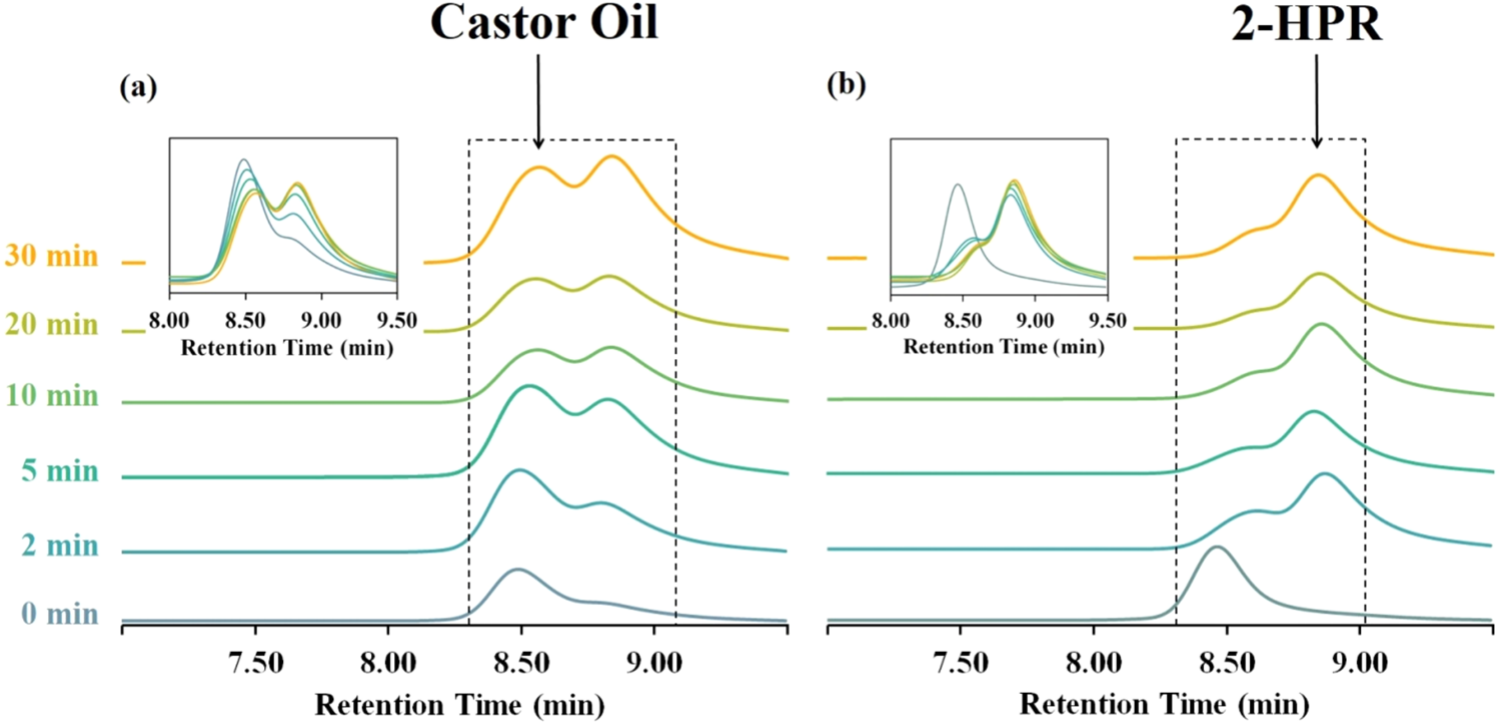
(a) The HPLC reaction investigation for the equimolar reaction mixture (glycerol ester bonding:propylene glycol 1:1). (b) The HPLC reaction investigation for the 100% excess reaction mixture (glycerol ester bonding:propylene glycol 1:2).

**Figure 7:**
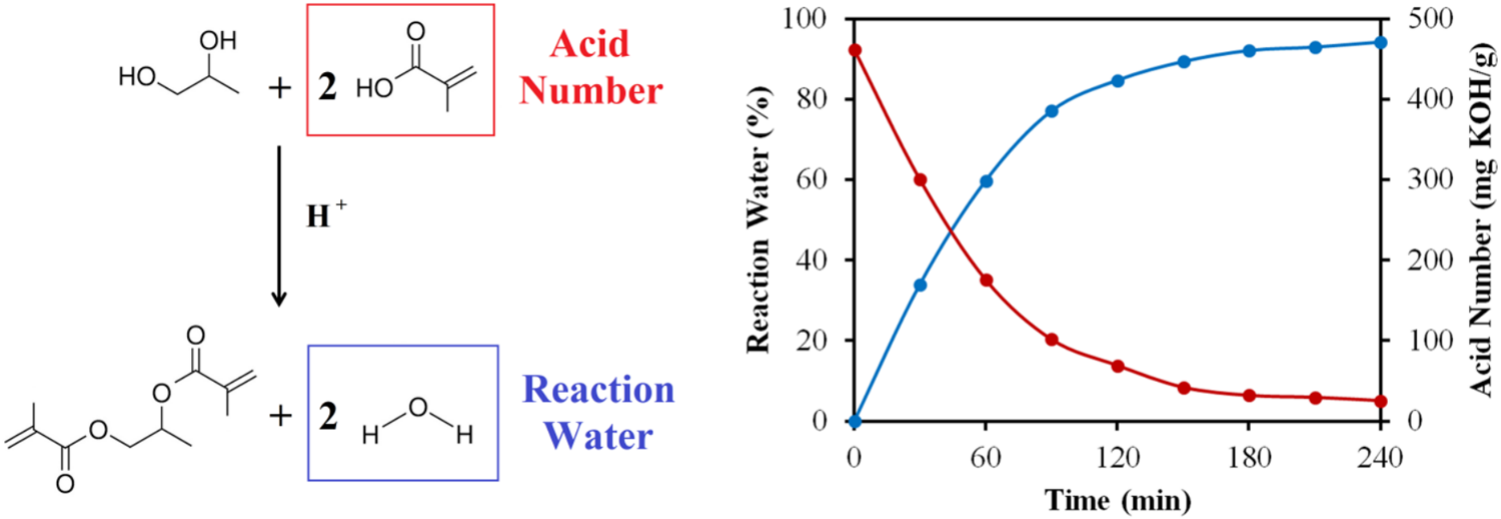
Propylene glycol dimethacrylate (PGDMA) synthesis reaction scheme, highlighting the decreasing acid number and increasing reaction water content during Fisher esterification. The reaction water quantification and the acid number measurements during the esterification.

The ^1^H NMR spectra confirming the synthesized compounds’ structure are illustrated in Figure 8. Next to 2-HPR (Figure S1) and PGDMA (Figure 8c) synthesis, we did not report 2-HPR methacrylation progress, producing 2-HPRDM (Figure 8a) since this study was provided in our previous publication, using GC-FID to analyze the decreasing methacrylic anhydride’s content and the increasing methacrylic acid’s quantity.^51^ The provided NMR spectra, in combination with other verification analyses, confirm the proposed structures of the obtained molecules based on the specific signals at selected chemical shift intervals. The presence of the methacrylate functional group is a key finding, confirming the curability of the produced compounds. Both eventual resin-forming products, 2-HPRDM and PGDMA, contain the reactive double bond signals in the chemical shift interval of 6.3–5.7 ppm (see Figure 8a,c). Additionally, the quantitative peak integrations confirm that the hydrogen sum corresponds to the proposed structures. ^1^H NMR, using chloroform-d, is not effective in analyzing protons in free hydroxyl groups (especially in lipids such as castor oil), as reported in many published articles.^52,53^ Therefore, FTIR and ESI-MS analyses were provided (see Supporting Information), confirming the appropriate *m*/*z* values (ESI-MS) and revealing the present −OH vibration and rotation signal (FTIR). This cross-analysis combination provides sufficient information for the structural identification.

**Figure 8:**
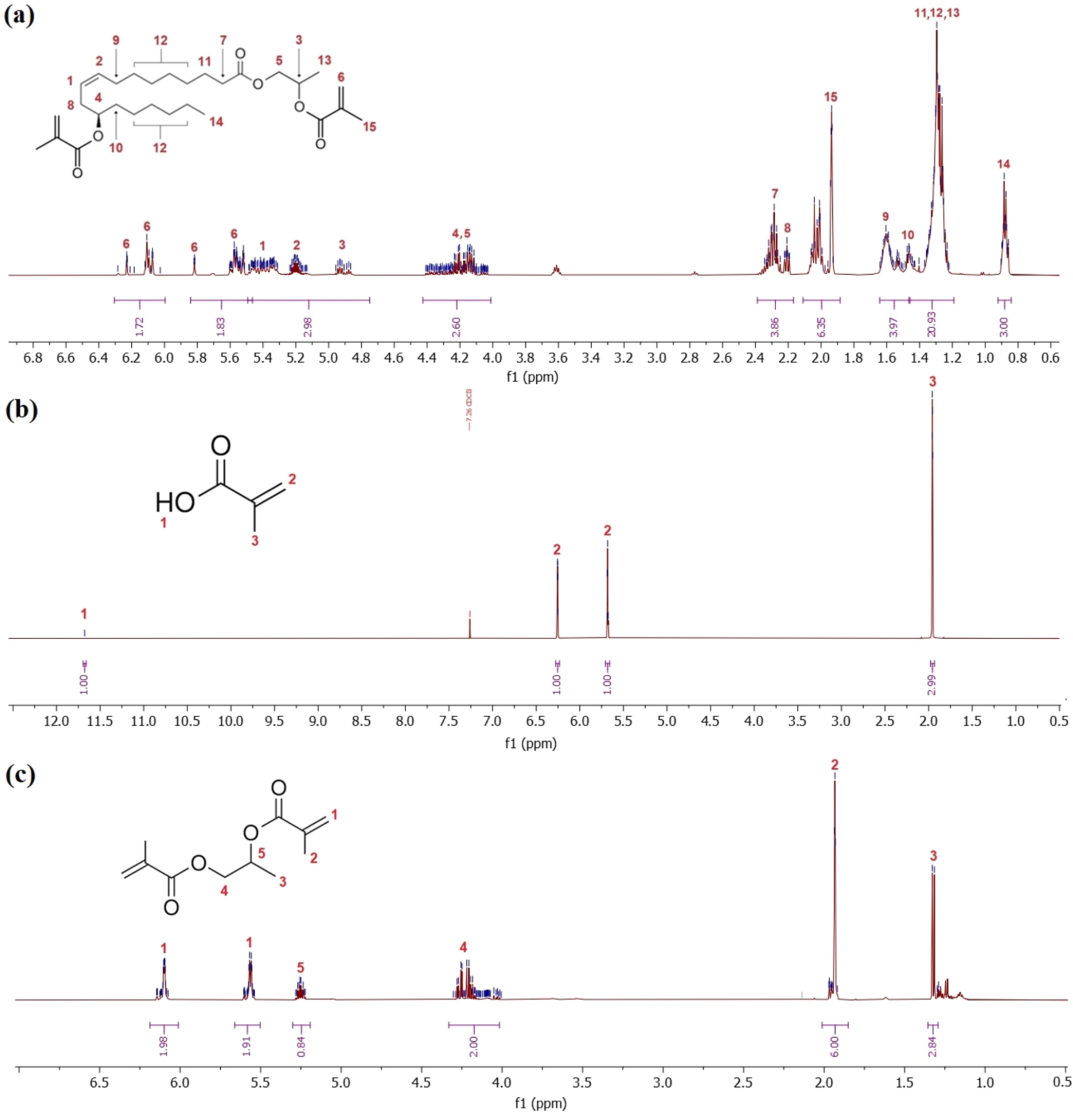
(a) ^1^H NMR spectrum of 2-hydroxypropyl ricinoleate dimethacrylate (2-HPRDM), (b) ^1^H NMR spectrum of the distilled methacrylic acid (MA) from 2-hydroxypropyl ricinoleate methacrylation, and (c) ^1^H NMR spectrum of propylene glycol dimethacrylate (PGDMA).

### 3.2 Mechanical Analysis of Formulated Resins

Dynamic mechanical analysis was chosen to investigate the mechanical properties of the formulated curable system at varying temperatures. Together with the mechanical investigation, the glass-transition temperatures (*T*_g_) and the cross-linking densities (ν_e_) were determined from DMA. Generally, photocurable systems based on modified oil structures tend to exhibit insufficient mechanical properties due to their low storage modulus, glass-transition temperature, and cross-linking density values.^56,57^ The novel synthesized compound, 2-hydroxypropyl ricinoleate dimethacrylate, possesses a chain-extending structure with a long linear carbon backbone between polymerizable methacrylate groups. The added propylene glycol dimethacrylate is a small molecule with two reactive groups, which can ensure a highly cross-linked molecular structure, boosting the mechanical properties of the eventual thermoset. The measured storage modules (*E*′), loss modules (*E*″), and calculated tan δ (−) are displayed in Figure 9.

**Figure 9:**
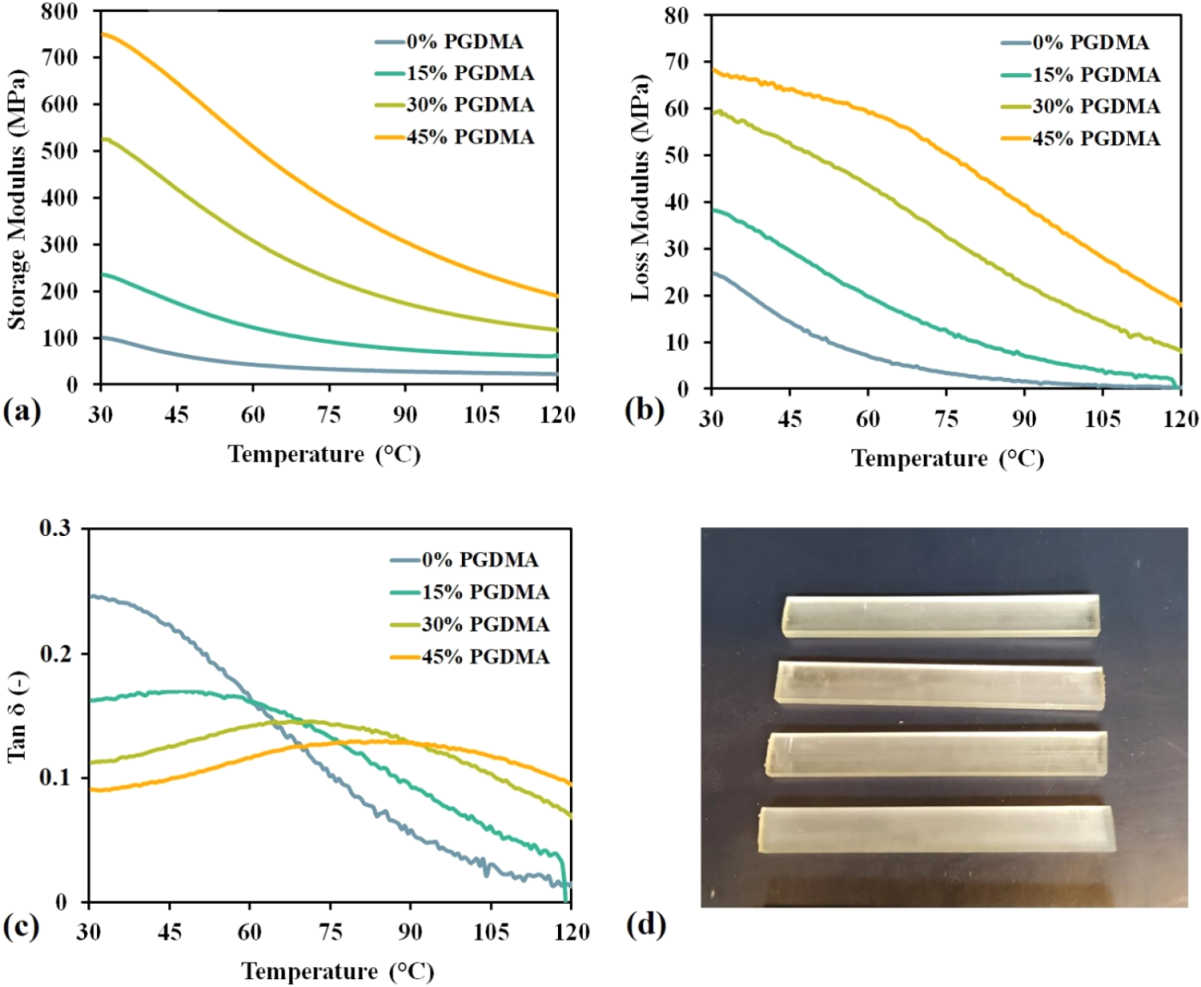
(a) The storage modulus (*E*′) measurements, (b) the loss modulus (*E*″) measurements, (c) the tan δ results, and (d) the used specimens for DMA analysis.

The positive effect on the thermomechanical properties of 2-HPRDM with the increasing PGDMA content is evident from the obtained DMA results. As Table 1 summarizes, all observed parameters determined and calculated from DMA continually increase with the cross-linker content in the cured resin. The pure 2-HPRDM (marked as 0% PGDMA) reached a storage modulus of 100.3 MPa, a glass-transition temperature below 30 °C, and a cross-linking density of 4.1 kmol/m^3^. These results are comparable to the acrylated soybean oil values (*E*′ of 303 MPa, *T*_g_ between 14 and 35 °C, and cross-linking density of 16.0 kmol/m^3^), where the cross-linking density is high. At the same time, the storage modulus remains lower due to the higher functionality of the modified triacylglyceride (greater than three) compared to 2-HPRDM.^57^ The photocurable system with the highest cross-linker content (45% PGDMA) reached *E*′ of 750.2 MPa, *T*_g_ of 68.3 °C, and a cross-linking density of 18.9 kmol/m^3^. In addition to its novelty and sustainable production, the recorded parameters from DMA exhibit exceptional potential compared to similarly derived oil-curable thermosets from the literature.^58,59^ In summary, the continually synthesized 2-HPRDM and PGDMA promise an innovative, sustainable approach to produce reactive biobased compounds using methacrylic acid instead of more expensive alternatives (alkyl halides and anhydrides), generating zero waste during the process and providing a competitive precursor system for material segments such as additive manufacture.

**Table 1:** Results of Dynamic Mechanical Analysis

dynamic mechanical analysis				
system composition	*E*′_30°C_ (MPa)	*E*″_30°C_ (MPa)	*T*_g_ (°C)	ν_e_ (kmol/m^3^)
0% PGDMA	100.3	24.7	<30.0	4.1
15% PGDMA	235.3	38.3	50.0	8.3
30% PGDMA	525.5	59.1	71.8	13.4
45% PGDMA	750.2	68.3	85.6	18.9

To determine the mechanical properties of prepared materials and to highlight differences in behavior with the addition of PGDMA, a tensile test and a flexural test were performed. The tensile test (see Table 2) aimed to determine the tensile strength of the material. In Figure 10a, the measured values for individual materials, ranging from 5.5 ± 0.4 to 16.1 ± 2.4 MPa, can be observed. The highest tensile strength exhibited a sample containing 45% PGDMA with 16.08 ± 2.44 MPa, in contrast to a sample with 0% PGDMA, which had the lowest (5.5 ± 0.4 MPa). The measured data indicate that the tensile strength increases with higher PGDMA content. The same trend was observed in the flexural test (see Table 3), which was also aimed at assessing flexural strength, where the measured values ranged from 5.1 ± 0.1 to 14.3 ± 1.0 MPa (as can be seen in Figure 10b). In this case, as well, the sample with 45% PGDMA exhibited the highest flexural strength, while the sample without PGDMA showed the lowest. Nevertheless, in both cases, the addition of just 15% PGDMA did not result in a significant increase in strength; therefore, this amount has no substantial effect on the tensile and flexural strengths.

**Figure 10:**
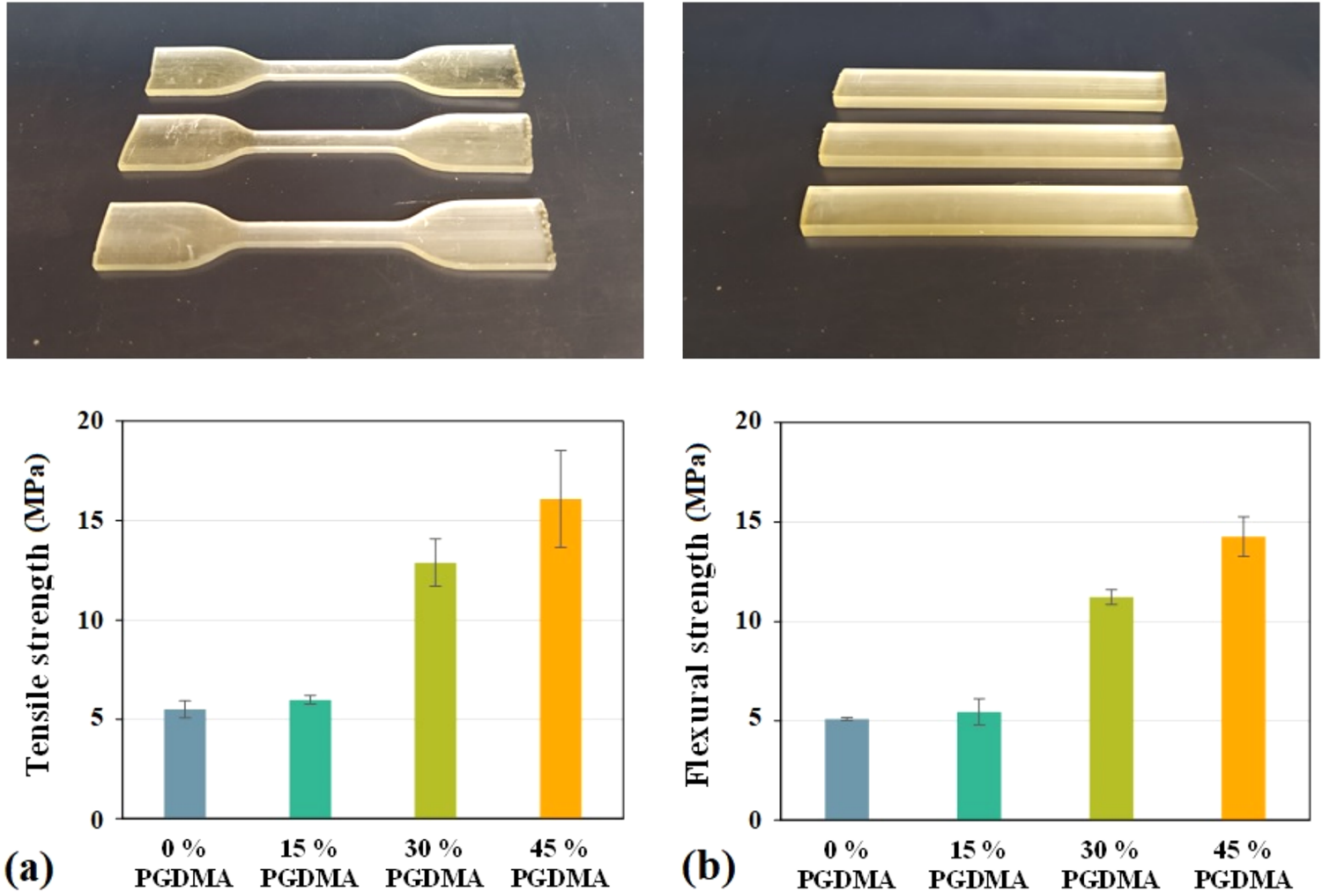
Measured specimens for the tensile and flexural tests. (a) The tensile test results. (b) The flexural test results.

**Table 2:** Results of the Tensile Test

tensile test								
	0% PGDMA		15% PGDMA		30% PGDMA		45% PGDMA	
	*E*_t_ (MPa)	σ_t_ (MPa)	*E*_t_ (MPa)	σ_t_ (MPa)	*E*_t_ (MPa)	σ_t_ (MPa)	*E*_t_ (MPa)	σ_t_ (MPa)
	105	4.9	315	6.2	619	12.4	1053	13.7
	111	5.4	286	5.8	639	14.0	1020	15.1
	109	6.0	306	5.8	680	11.0	1023	17.1
	114	5.7	307	5.9	636	13.5	1024	19.8
	111	5.5	298	6.2	632	13.6	1024	14.7
mean	110	5.5	302	6.0	641	12.9	1029	16.1
SD	3	0.4	11	0.2	23	1.2	14	2.4

**Table 3:** Results of the Flexural Test

flexural test								
	0% PGDMA		15% PGDMA		30% PGDMA		45% PGDMA	
	*E*_f_ (MPa)	σ_f_ (MPa)	*E*_f_ (MPa)	σ_f_ (MPa)	*E*_f_ (MPa)	σ_f_ (MPa)	*E*_f_ (MPa)	σ_f_ (MPa)
	110	5.2	219	5.7	538	11.7	940	13.7
	102	5.1	236	4.3	538	11.1	910	13.9
	111	5.0	235	5.5	516	10.8	902	16.0
	105	5.1	232	6.0	498	11.4	914	13.6
	106	5.3	225	5.8	-	-	902	14.1
mean	110	5.1	302	5.5	641	11.2	1029	14.3
SD	3	0.1	11	0.7	23	0.4	14	1.0

Based on the measured values, it can be observed that the added reactive diluent has a positive effect on the resulting mechanical strength of the material, particularly in terms of tensile and flexural strengths. It should also be noted that a noticeable positive impact was observed starting from the addition of 30% PGDMA. The primary reason for the observed increase in strength with higher PGDMA content is the greater degree of cross-linking. As the amount of PGDMA in the sample increases, so does the concentration of reactive double bonds that participate in the curing process. Since PGDMA is a bifunctional molecule containing two methacrylate groups, it can form more cross-links between chains, leading to a denser network structure. As a result, the mechanical strength improved. However, the increased cross-linking density also makes the material more brittle.

### 3.3 The Printability of the Synthesized System

Additive manufacturing is shifting toward biobased alternatives to entirely artificial curable precursors due to cost optimization, environmentally friendly system application, and legislative requirements.^60^ In this article, we propose a sustainable, wasteless, and efficient production of a reactive biobased system, mechanically enhanced with an innovatively synthesized cross-linker. The most optimal mixture containing 45 wt % of the cross-linker was fabricated via mSLA 3D printing to verify its suitability for this material application. The resulting prototype is illustrated in Figure 11. The presented images reflect a highly detailed prototype fabrication investigated by using optical microscopy. Based on both the instrumentation specification and the observed prototype, the resolution limit for the particular mSLA print is 0.01 mm. Our selected resolution level was set to 50 μm. At this cured layer thickness, the used irradiation source in the mSLA printer (possessing 990 mW/m^2^) reached a cuing depth of >99% (for an irradiation source of such power, a much thicker printed layer would reach quantitative cure at the set exposure time). The presented photos confirm the appropriate reactivity and mechanical property profile of the proposed reactive system for additive manufacture. Next to the detailed object production, all of the thermomechanical investigation specimens (see Figure 9d) and the tensile/flexural test specimens (see Figure 10) were produced by mSLA 3D printing.

**Figure 11:**
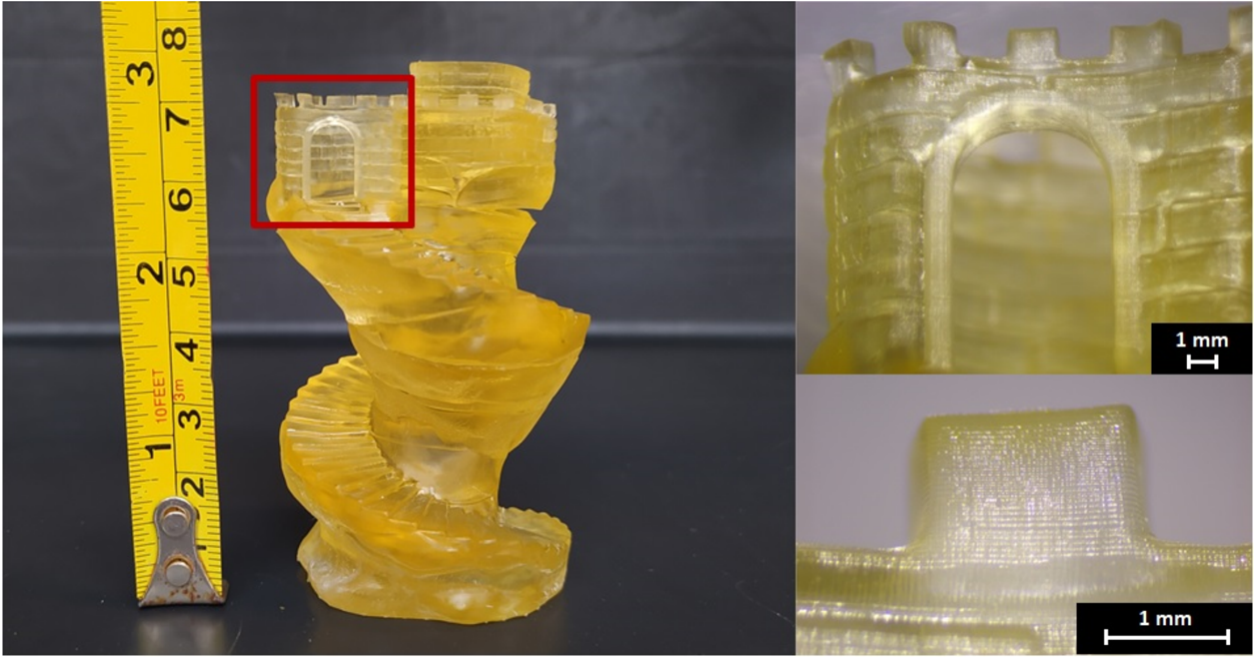
3D-printed prototype from the synthesized system containing 2-hydroxypropyl ricinoleate dimethacrylate (2-HPRDM) and 45 wt % of the cross-linker propylene glycol dimethacrylate (PGDMA).

## 4 Conclusions

This article evaluates a synthesis route leading to a novel compound, 2-hydroxypropyl ricinoleate dimethacrylate (2-HPRDM). The described approach involves a study of castor oil’s transesterification, focusing on the differences between equimolar and excess amounts of the used reacting diol, propylene glycol (PG). As the HPLC-monitored study revealed, a reacting excess of PG is necessary for quantitative transesterification. The ricinoleic acid propylene glycol ester methacrylation followed the castor oil’s transesterification, leading to the curable 2-hydroxypropyl ricinoleate dimethacrylate (2-HPRDM). Methacrylic anhydride served as an acyl donor for this reaction, producing methacrylic acid (MA) during the reaction. This secondary product was separated via distillation and used for propylene glycol dimethacrylate (PGDMA) synthesis. The entire synthesis approach utilized inexpensive catalysts (sodium hydroxide, potassium acetate, and sulfuric acid), ensuring sustainable character and minimizing waste. The syntheses yielded high yields (85–95%), and the synthesized compounds were structurally characterized by NMR, ESI-MS, and FTIR. The material performance was investigated via DMA, tensile, and flexural tests. The PGDMA increasing content in the curable resin enhanced the eventual material’s properties. The best-performing system achieved a storage modulus of 750 MPa, a glass-transition temperature of 85.6 °C, a cross-linking density of 18.9 kmol/m^3^, a tensile strength of 16.1 ± 2.4 MPa, and a flexural strength of 14.3 ± 1.0 MPa. Eventually, the best-performing system was verified as a suitable precursor for additive manufacturing.

## Supplementary Material



## Notes

Structural characterizations of the synthesized products (Figures S1–S12) and the tensile/flexural stress–strain curves (Figures S13 and S14) (PDF)
