# Supplementary material for: Innovative Castor Oil Derivative Synthesized through a Sustainable Approach Generating Reactive Cross-Linker from Secondary Products for Additive Manufacturing
Source: ACS Polym Au. 2025 Jul 30;5(5):545–56. doi: 10.1021/acspolymersau.5c00055 (PMC12511972; doi:10.1021/acspolymersau.5c00055)
Supplement: Supplementary file 1 [file lg5c00055_si_001.pdf]

Innovative Castor Oil Derivative Synthesized Through a Sustainable Approach Generating Reactive Crosslinker from Secondary Products for Additive Manufacturing

Vojtěch Jašek[a*], Veronika Lavrinčíková[a], Otakar Bartoš[a], Jan Prokeš[a], Radek Přikryl[a], and Silvestr Figalla[a]

a Institute of Materials Chemistry, Faculty of Chemistry, Brno University of Technology, 61200 Brno, Czech Republic.

*corresponding author: xcjasekv@vutbr.cz

Table of content

1. **Structural verification of the synthesized compounds**
   - **Figure S1-S3.** Cross-analysis of 2-hydroxypropyl ricinoleate (2-HPR)
   - **Figure S4-S6.** Cross-analysis of 2-hydroxypropyl ricinoleate dimethacrylate (2-HPRDM)
   - **Figure S7-S9.** Cross-analysis of distilled methacrylic acid (MA)
   - **Figure S10-S12.** Cross-analysis of propylene glycol dimethacrylate (PGDMA)
2. **Mechanical investigation data**
   - **Figure S13.** Tensile stress-strain curves.
   - **Figure S14.** Flexural stress-strain curves.

1. Structural verification of the synthesized compounds

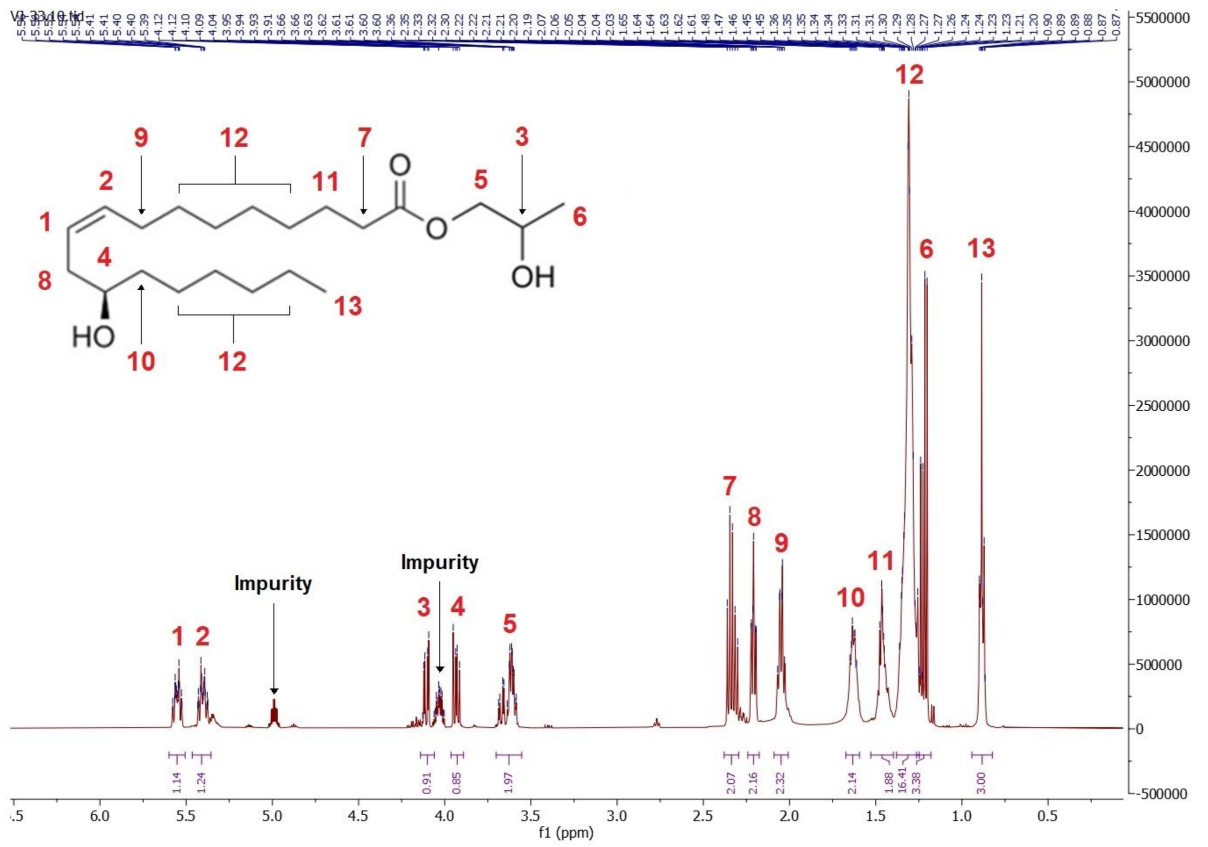

Figure S1. ^{1}H NMR spectrum of the synthesized 2-hydroxypropyl ricinoleate (2-HPR).

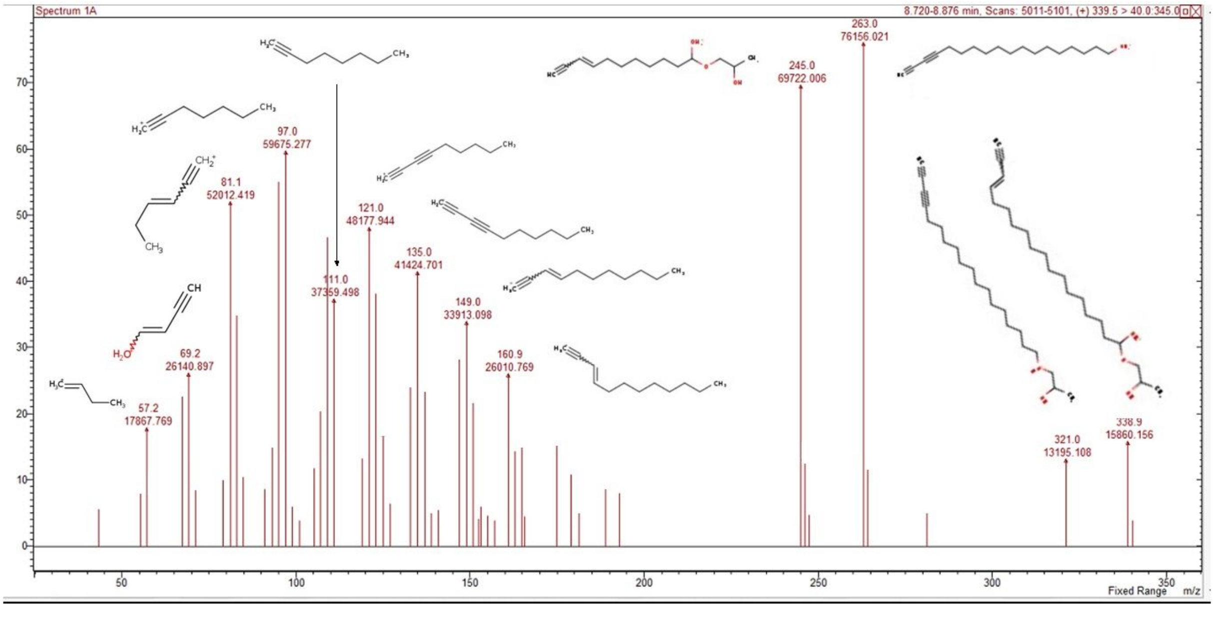

Figure S2. ESI-MS spectrum of the synthesized 2-hydroxypropyl ricinoleate (2-HPR).

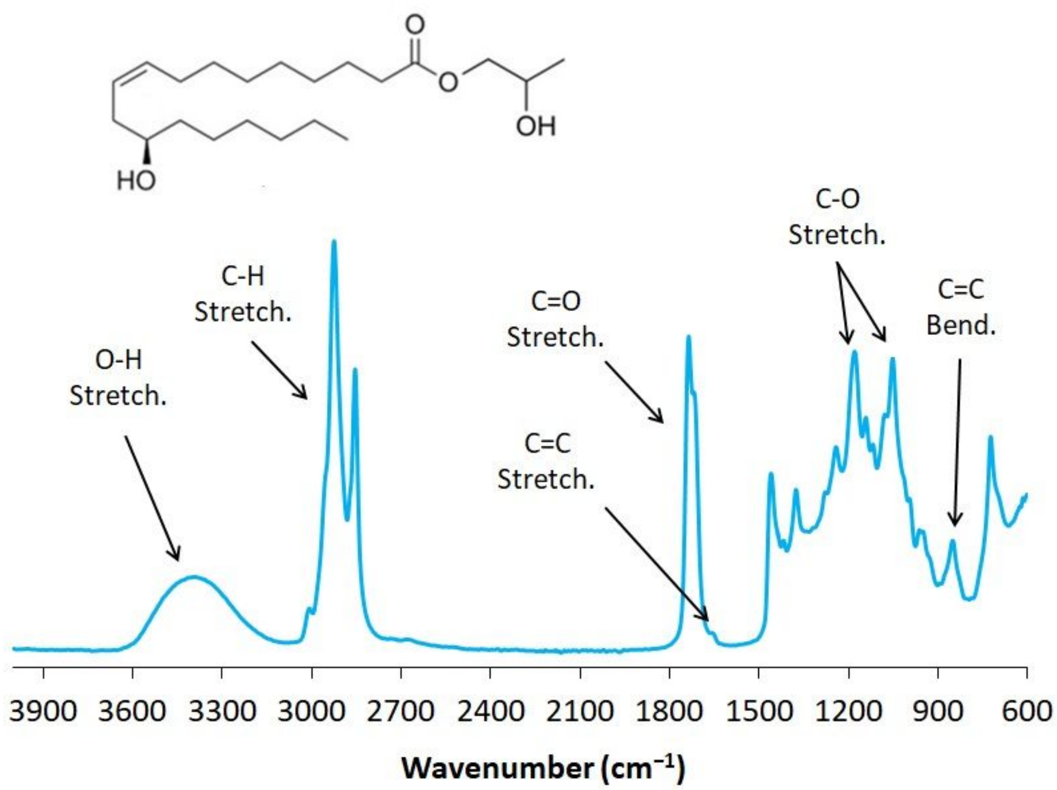

Figure S3. FT-IR spectrum of the synthesized 2-hydroxypropyl ricinoleate (2-HPR).

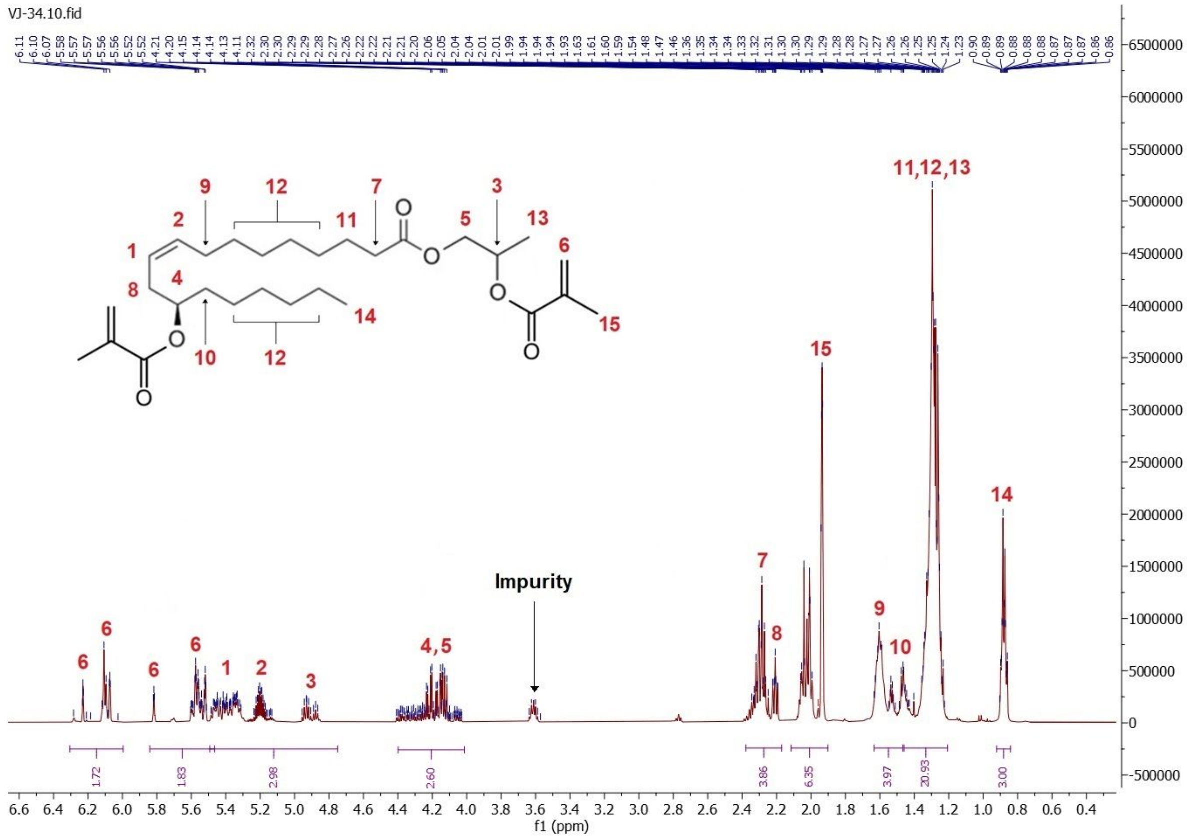

Figure S4. ¹H NMR spectrum of the synthesized 2-hydroxypropyl ricinoleate dimethacrylate (2-HPRDM).

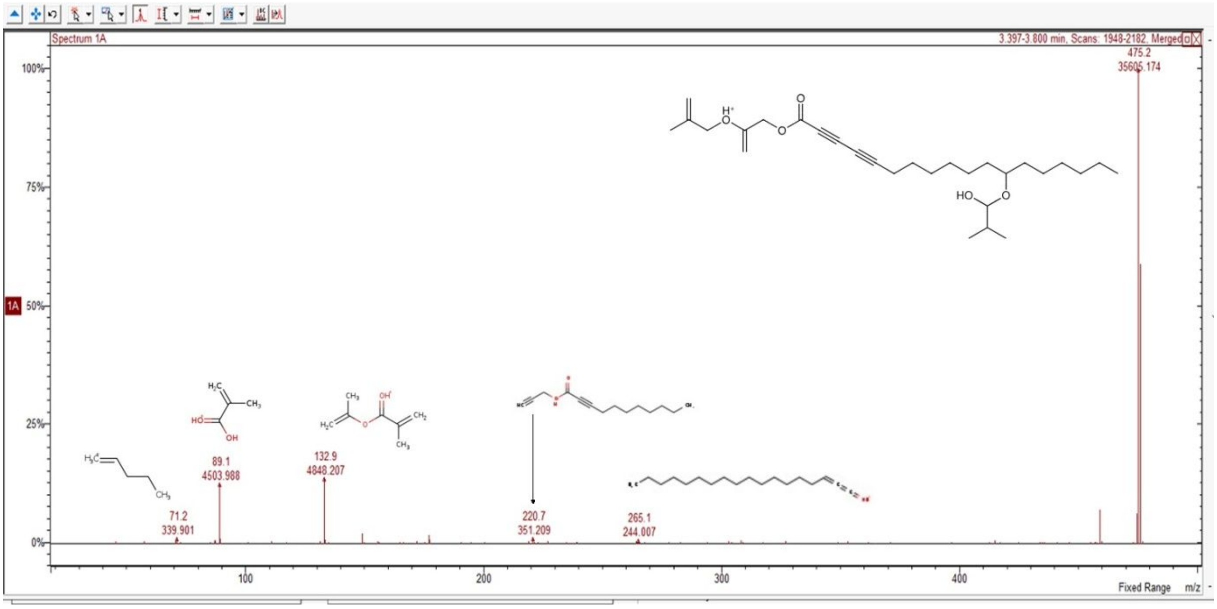

Figure S5. ESI-MS spectrum of the synthesized 2-hydroxypropyl ricinoleate dimethacrylate (2-HPRDM).

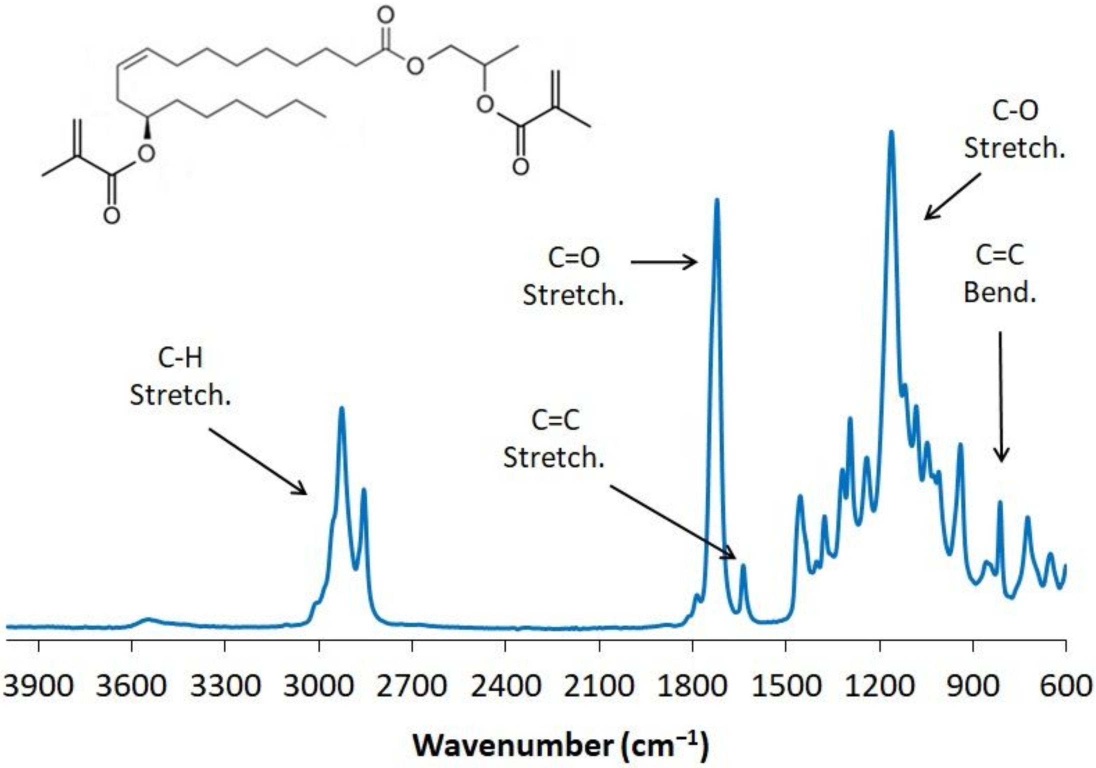

Figure S6. FT-IR spectrum of the synthesized 2-hydroxypropyl ricinoleate dimethacrylate (2-HPRDM).

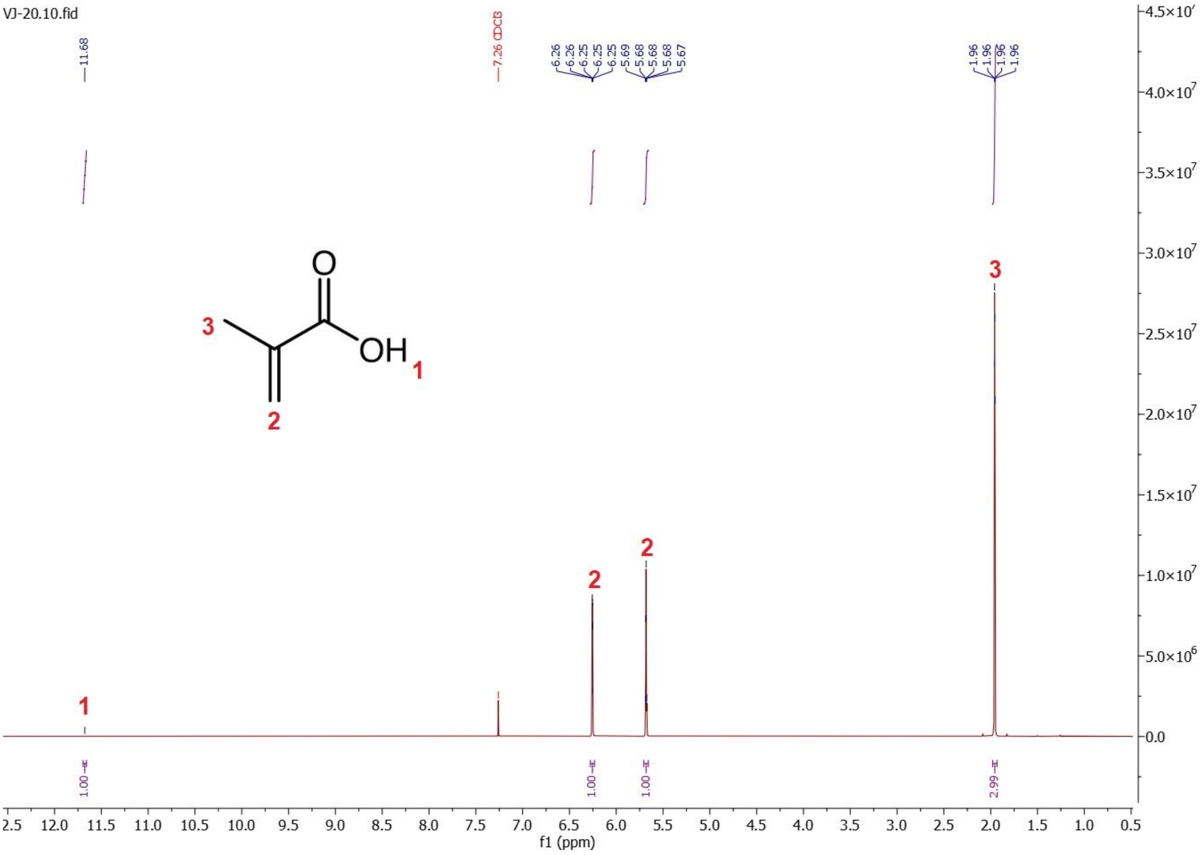

Figure S7. ^{1}H NMR spectrum of the distilled methacrylic acid (MA).

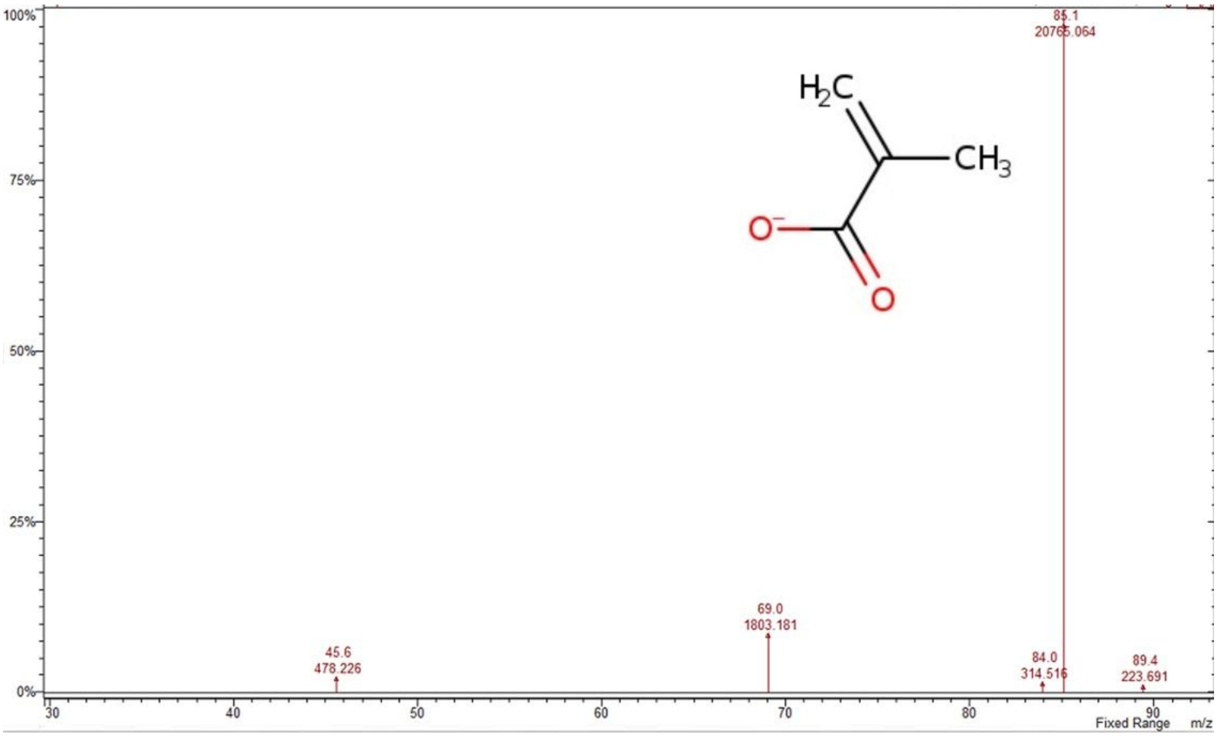

Figure S8. ESI-MS spectrum of the distilled methacrylic acid (MA).

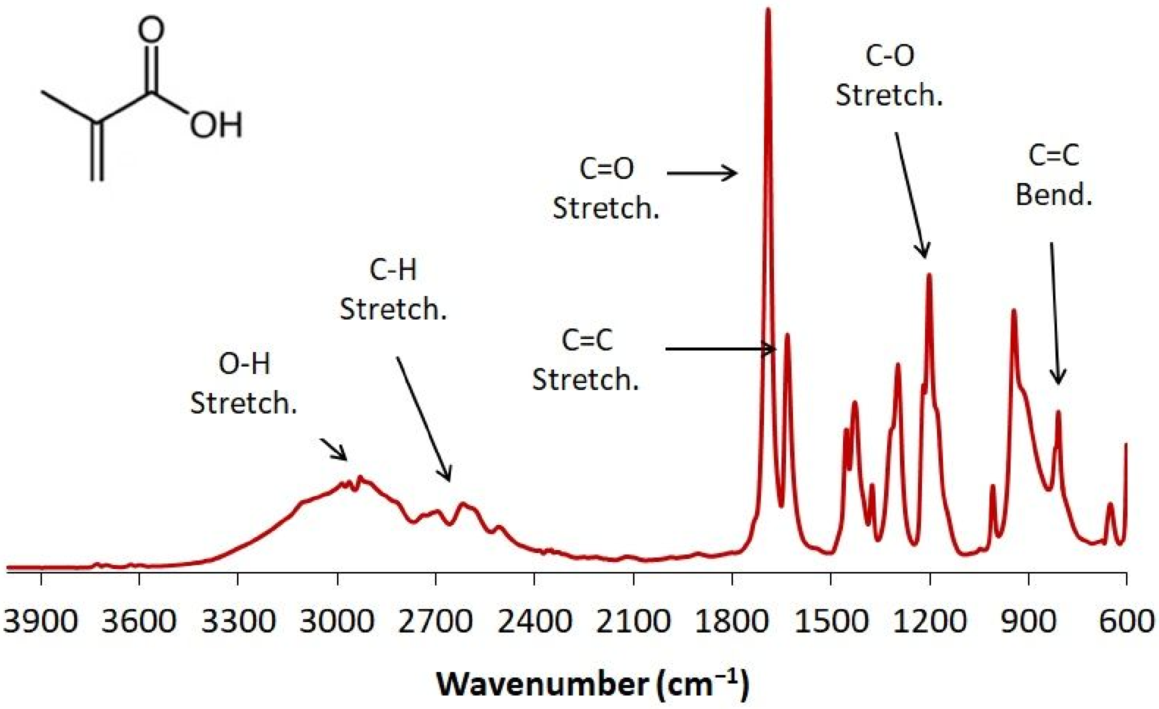

Figure S9. FT-IR spectrum of the distilled methacrylic acid (MA).

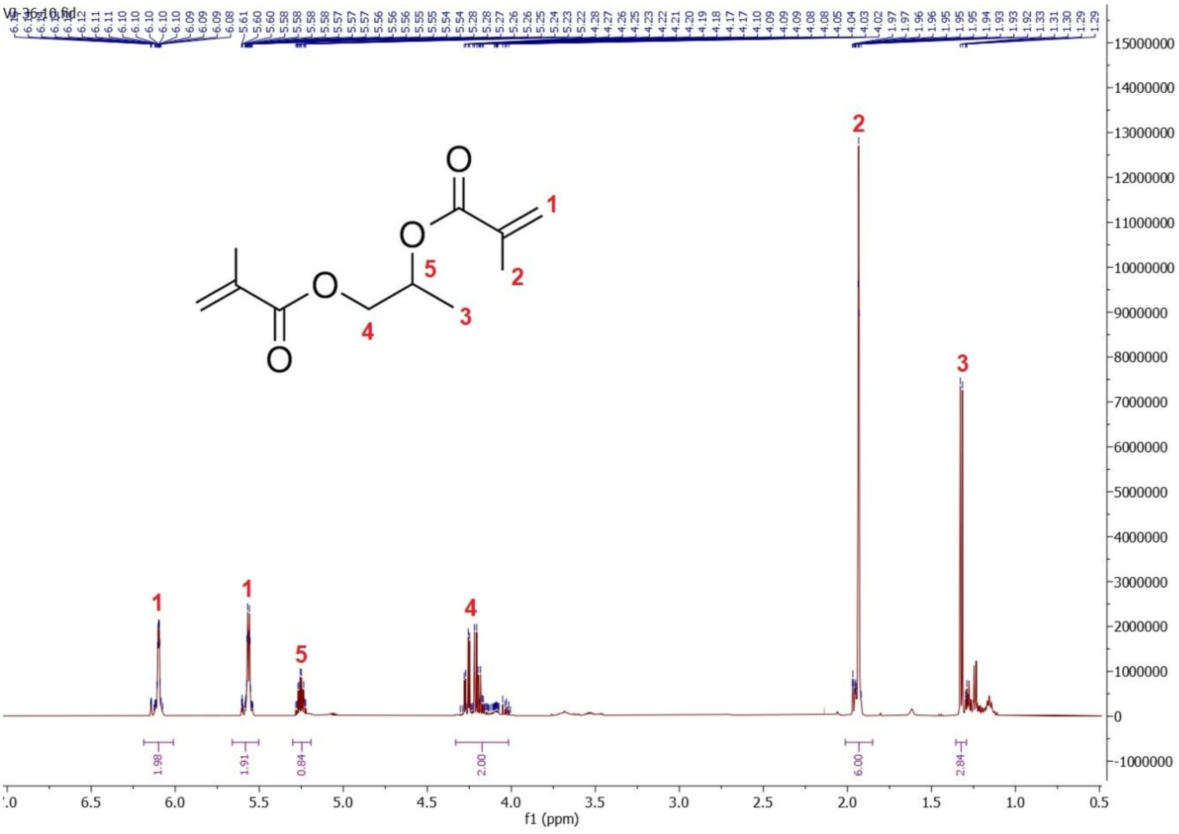

Figure S10. ^{1}H NMR spectrum of the synthesized propylene glycol dimethacrylate (PGDMA).

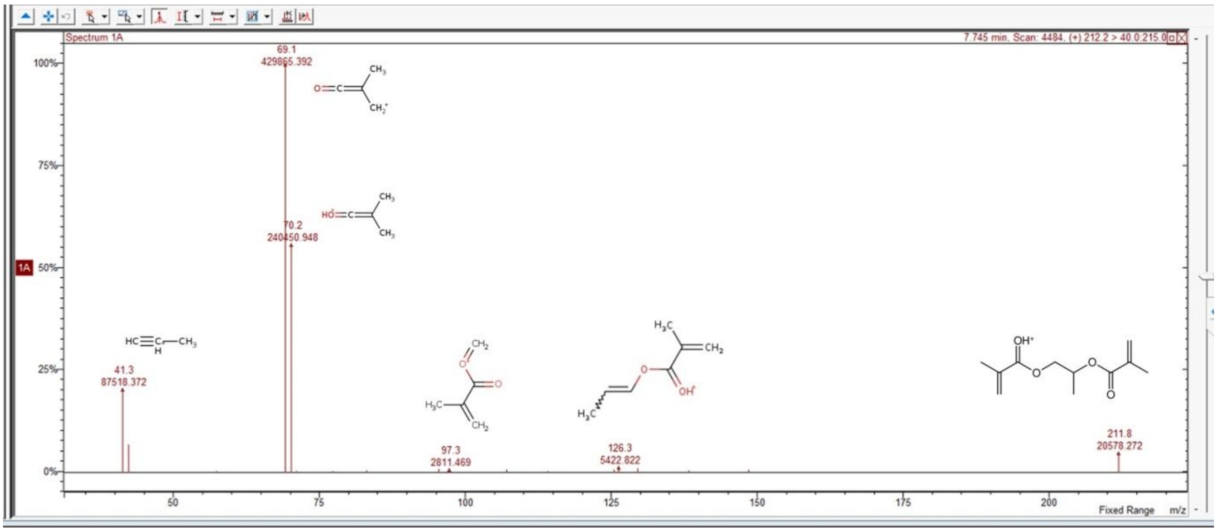

Figure S11. ESI-MS spectrum of the synthesized propylene glycol dimethacrylate (PGDMA).

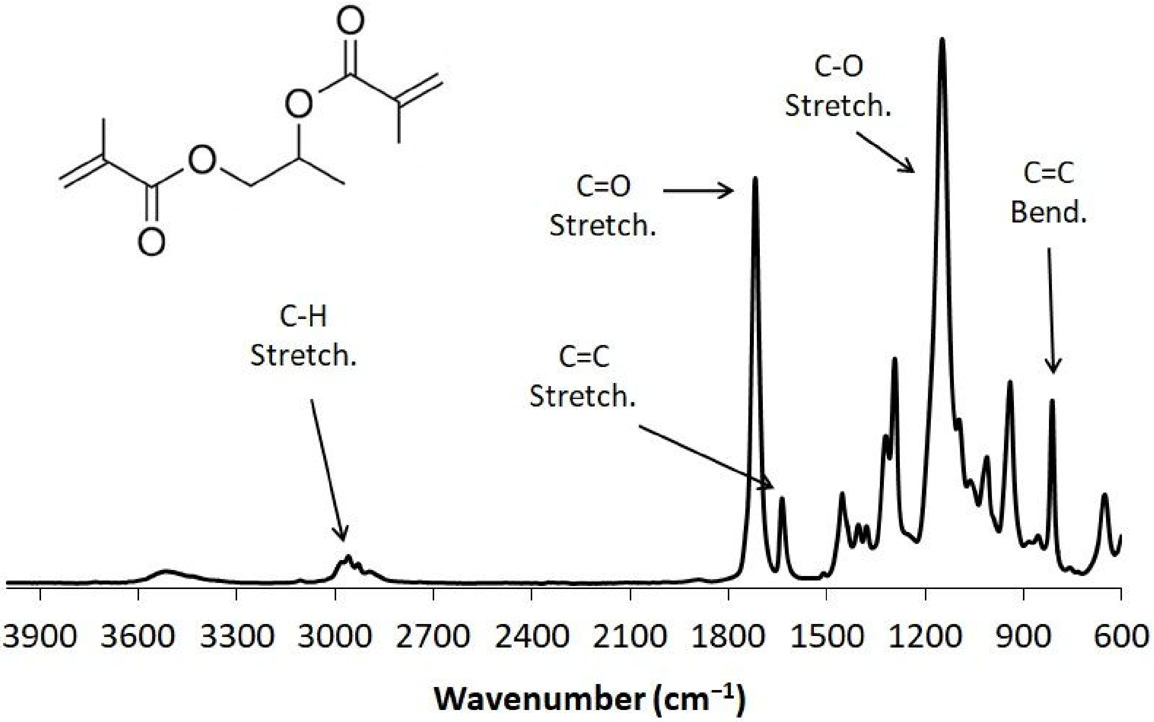

Figure S12. FT-IR spectrum of the synthesized propylene glycol dimethacrylate (PGDMA).

2. Mechanical investigation data

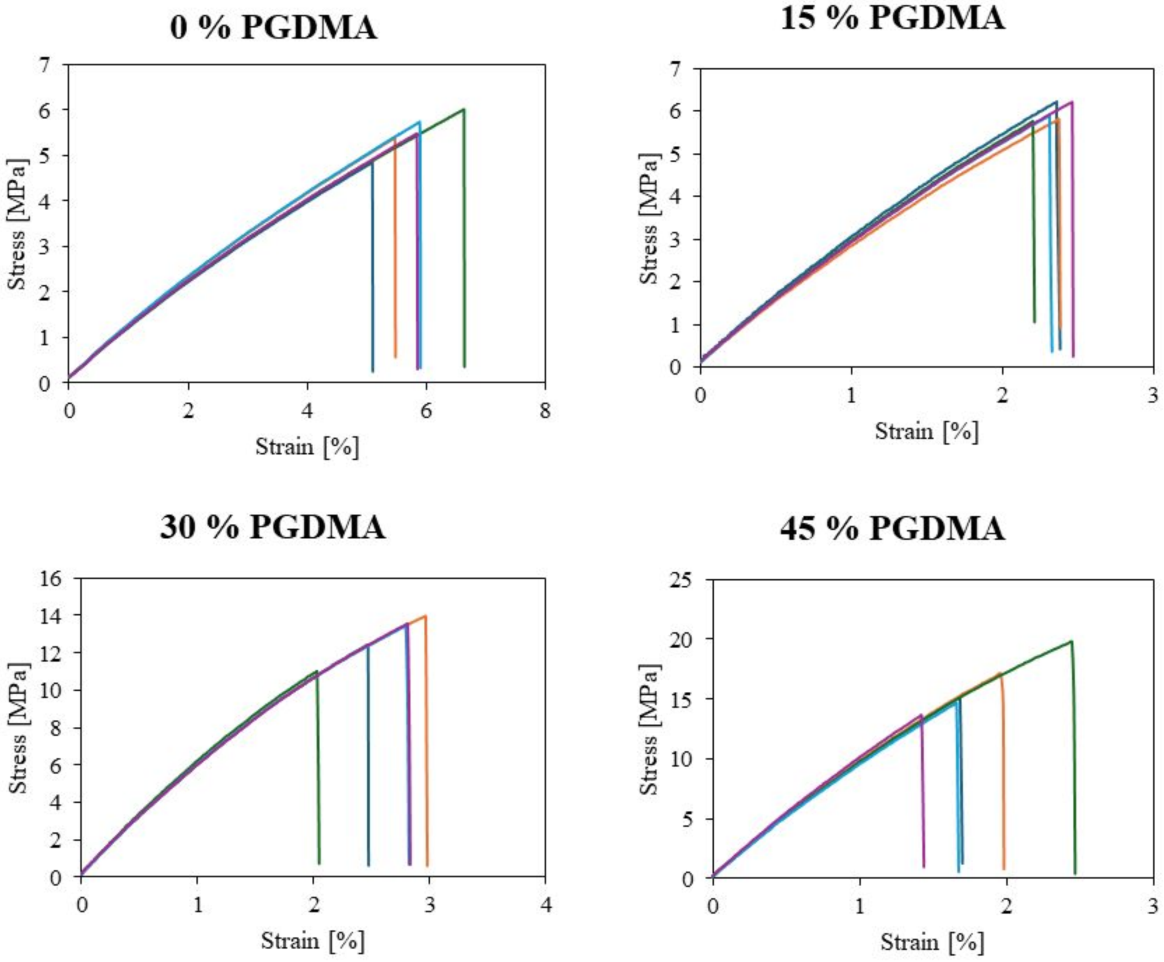

Figure S13. The tensile stress-strain curves of the cured formulated 3D-printed resins.

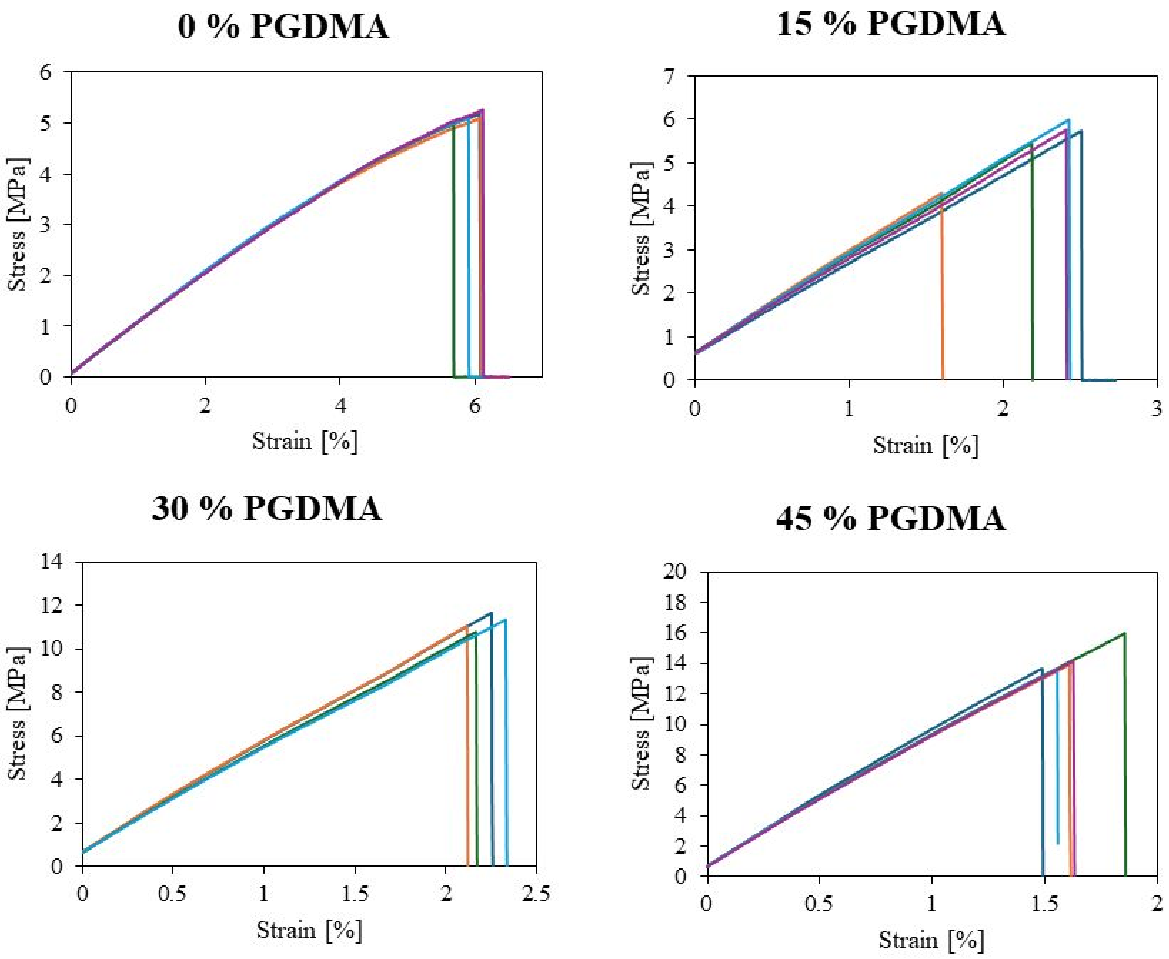

Figure S14. The flexural stress-strain curves of the cured formulated 3D-printed resins.
